# Bridging Classical and Revised Reinforcement Sensitivity Theory Research: A Longitudinal Analysis of a Large Population Study

**DOI:** 10.3389/fpsyg.2021.737117

**Published:** 2021-12-20

**Authors:** Daniela A. Espinoza Oyarce, Richard Burns, Peter Butterworth, Nicolas Cherbuin

**Affiliations:** Centre for Research on Ageing, Health & Welbeing, Research School of Population Health, The Australian National University, Canberra, ACT, Australia

**Keywords:** reinforcement sensitivity theory, factor analysis, measurement invariance, longitudinal, neuropsychology

## Abstract

The reinforcement sensitivity theory (RST) proposes that neurobiological systems mediate protective and appetitive behaviours and the functioning of these systems is associated to personality traits. In this manner, the RST is a link between neuroscience, behaviour, and personality. The theory evolved to the present revised version describing three systems: fight-flight-freezing, behavioural approach/activation (BAS), and behavioural inhibition (BIS). However, the most widely available measure of the theory, the BIS/BAS scales, only investigates two systems. Using a large longitudinal community survey, we found that the BIS/BAS scales can be re-structured to investigate the three systems of the theory with a BIS scale, three BAS scales, and a separate fight-flight-freezing system (FFFS) scale. The re-structured scales were age, sex, and longitudinally invariant, and associations with personality and mental health measures followed theoretical expectations and previously published associations. The proposed framework can be used to investigate behavioural choices influencing physical and mental health and bridge historical with contemporary research.

## Introduction

The reinforcement sensitivity theory (RST) is one of the most influential behavioural models of the 20th century. The RST was developed from empirical data on the neurobiology of anxiety (Gray, 1970, 1982; Gray and McNaughton, 2000) and provides a unique link between brain and behaviour. Classical RST describes behaviour as the interrelation of three systems: the behavioural inhibition (BIS), behavioural approach/activation (BAS), and fight-flight (FFS). The BIS includes the hippocampus and closely connected structures and is a global avoidance system that inhibits ongoing behaviour in novel, uncertain or dangerous conditions to increase risk assessment and attention. Punishment and fear activate this system and BIS functioning is associated with anxiety (Gray, 1970, 1982; Corr et al., 1995; Corr, 2004; Corr and Perkins, 2006; Smillie et al., 2006b). The BAS comprises reward pathways and is a global approach system that directs behaviour towards a goal or reward, increasing exploration to ensure biological needs are met. Consequently, rewards activate this system and BAS functioning is associated with impulsivity (Corr et al., 1995; Pickering and Gray, 1999; Gray and McNaughton, 2000; Corr, 2004; Corr and Perkins, 2006; Smillie et al., 2006b). Finally, the FFS includes midbrain structures and is a secondary avoidance system that mediates fight-or-flight behaviour. Pain activates this system and FFS functioning is associated with anger and panic (Corr et al., 1995; Corr, 2004; Corr and Perkins, 2006; Smillie et al., 2006b) but these associations were less clear than for the BIS and BAS (Carver and White, 1994; Smillie et al., 2006b). From the descriptions above, the link between brain and behaviour also extends to personality and, in this manner, the RST is a model that investigates current and future behaviour and can be utilised to understand physical and mental health outcomes.

Considering the potential of the RST, a number of questionnaires were developed to estimate BIS and BAS sensitivities. The most widely used measure are the BIS/BAS scales (Carver and White, 1994), which have been used to investigate sensitivities to these systems and to demonstrate substantive associations to personality and health. For instance, the BAS has been shown to be positively associated to impulsivity (Poythress et al., 2008; Beck et al., 2009) and positive affect (Jorm et al., 1999; Smillie et al., 2006a), and impulsivity-related behaviours such as aggression and delinquency (Yu et al., 2011), smoking uptake (Baumann et al., 2014), drug and alcohol use (Johnson et al., 2003; Wardell et al., 2011), and binge/purge eating disorders (Beck et al., 2009). On the other hand, the BIS has been shown to be positively associated to neuroticism (Jorm et al., 1999; Heym et al., 2008; Yu et al., 2011) and negative affect (Jorm et al., 1999), and neuroticism-related disorders such as anxiety (Jorm et al., 1999; Johnson et al., 2003; Poythress et al., 2008) and depression (Jorm et al., 1999; Johnson et al., 2003). Emerging data on fear, anxiety, and reward behaviour, however, led to a major revision of the RST (Gray and McNaughton, 2000).

In the revised RST, the BIS and BAS are reconceptualised and FFS redefined into the fight-flight-freezing system (FFFS). The revised BIS is a supervisory system that resolves conflicts when approach and avoidance behaviours are concurrently engaged, or when competing goals or competing avoidance actions are possible. The system inhibits ongoing behaviour during conflicts and increases risk assessment, attention, and cautious approach to promote a single response. BIS activation during conflict resolution is associated with anxiety with inability to resolve conflicts associated with increased levels of anxiety (Gray and McNaughton, 2000; Corr, 2004; Corr and Perkins, 2006; Smillie et al., 2006b). The revised BAS remains a global approach system but BAS functioning is multidimensional and associated with both impulsivity and extraversion (Corr, 2004; Corr and Perkins, 2006; Smillie et al., 2006a,b). Finally, the FFFS is a global avoidance and escape system in threatening conditions and promotes fight, flight, or freezing behaviour depending on perceived risk. Specifically, high risk promotes attack, intermediate risk promotes flight or freezing, and low risk does not engage defensive behaviour. FFFS functioning is associated with fear and panic (Gray and McNaughton, 2000; Corr, 2004; Corr and Perkins, 2006; Smillie et al., 2006b).

The revision of the RST, however, was not supplemented with a concurrent update of psychometric tools. The first measure reflecting the new conceptualisation was developed 9 years after the revision of the theory with the Jackson-5 scales (Jackson, 2009), yet the constructs investigated did not follow theoretical associations and likely limited its use. Specifically, the BIS and BAS scales correlated positively despite the differing functions of these systems and the FFFS constructs did not converge into a single scale (Krupić et al., 2016a). A similar situation was observed for another measure, the RST questionnaire (Smederevac et al., 2014), and FFFS constructs. The recently developed revised RST questionnaire (Reuter et al., 2015) and RST personality questionnaire (Corr and Cooper, 2016) have addressed the methodological issues of the earlier measures, nevertheless there is no consensus on whether the BAS should be investigated using a single scale, as proposed by Smederevac et al. (2014) and Reuter et al. (2015), or multiple subscales, as proposed by Corr and Cooper (2016) and Krupić et al. (2016a,b). In this manner, newer measures are yet to be widely adopted and there is currently no robust way to link past research based on the BIS/BAS scales, which have been used for over 25 years and provide a wealth of behavioural and personality data. Indeed, previous attempts at linking past research with the BIS/BAS scales (Krupić et al., 2016a) did not address the conflation of fear and anxiety constructs in the BIS scale, which are based on the original conceptualisation.

Therefore, the aims of the present study were to investigate whether the earlier conceptualisation of the BIS based on the scales of Carver and White (1994), which includes items relating to both anxiety and fear, can also be used to additionally estimate the fear component of the FFFS. In addition, the study will also systematically investigate the stability of the BIS, BAS, and FFFS scales across different cohorts in terms of age, sex, and time using rich epidemiological data.

## Materials and Methods

### Participants and Procedure

Participants were drawn from the Personality and Total Health (PATH) Through Life Project, a longitudinal community-based sample from the cities of Canberra and neighbouring Queanbeyan, Australia (Anstey et al., 2012). Participants were randomly selected from these communities using electoral rolls, since voting is compulsory for all Australian citizens aged 18 and above. PATH included 7,485 participants at baseline, within three age cohorts: young (20–24 years), midlife (40–44 years), and old-age (60–64 years).

All participants provided written consent and were interviewed every 4 years either at The Australian National University or at their home, where they completed self-report questionnaires using personal computers (Anstey et al., 2012; Windsor et al., 2012). Each wave of data collection of the PATH project has been approved by the Human Research Ethics Committee of The Australian National University. The present study includes data from four waves of measurement from 1999 to 2001, 2003 to 2005, 2007 to 2010, and 2011 to 2014 (Table 1). Retention rates from baseline were 53% for young, 71% for midlife, and 64% for old-age cohorts; with a higher proportion of females in the young cohort and higher proportion of males in the old-age cohort after 12 years of follow-up (Anstey et al., 2021).

**Table 1:** Descriptive statistics by age and wave of measurement.

		Total				Young			Midlife			Old-age		
		*n* = 7,485				*n* = 2,404			*n* = 2,530			*n* = 2,551		
		*n*	*Mean*	*SD*	*α*	*n*	*Mean*	*SD*	*n*	*Mean*	*SD*	*n*	*Mean*	*SD*
Baseline age	(years)	7,484	43.47	16.29		2,404	23.11	1.48	2,530	43.13	1.45	2,550	63.00	1.47
Baseline female		3,813	50.9^†^			1,242	51.7^†^		1,337	52.8^†^		1,234	48.4^†^	
Baseline education	(years)	7,339	14.32	2.34		2,389	14.58	1.58	2,527	14.58	2.34	2,423	13.78	2.84
BIS	wave 1	7,424	20.44	3.39	0.77	2,384	20.46	3.63	2,507	20.69	3.34	2,533	20.18	3.18
	wave 2	6,647	20.50	3.40	0.78	2,122	20.71	3.67	2,341	20.67	3.40	2,184	20.12	3.10
	wave 3	6,065	20.51	3.49	0.77	1969	20.99	3.70	2,161	20.76	3.41	1935	19.74	3.23
	wave 4	4,588	20.48	3.50	0.77	1,214	21.32	3.67	1778	20.68	3.48	1,596	19.61	3.18
BAS-d	wave 1	7,428	10.26	2.52	0.80	2,383	11.16	2.31	2,510	10.04	2.44	2,535	9.63	2.54
	wave 2	6,654	10.22	2.51	0.82	2,126	11.16	2.35	2,340	10.06	2.40	2,188	9.48	2.48
	wave 3	6,076	10.02	2.55	0.81	1970	10.86	2.45	2,163	9.83	2.44	1943	9.40	2.56
	wave 4	4,604	9.84	2.49	0.79	1,215	10.51	2.43	1781	9.75	2.35	1,608	9.43	2.58
BAS-f	wave 1	7,428	11.17	2.32	0.72	2,384	12.35	2.06	2,510	10.91	2.11	2,534	10.33	2.29
	wave 2	6,658	11.15	2.26	0.73	2,126	12.21	2.11	2,341	10.93	2.13	2,191	10.36	2.16
	wave 3	4,095	10.58	2.24	0.69	*NA* ^‡^	*NA* ^‡^	*NA* ^‡^	2,151	10.80	2.22	1944	10.34	2.24
	wave 4	4,604	10.70	2.23	0.68	1,214	11.28	2.21	1782	10.57	2.16	1,608	10.39	2.25
BAS-r	wave 1	7,428	16.67	2.09	0.70	2,383	17.30	1.92	2,511	16.48	2.04	2,534	16.28	2.15
	wave 2	6,653	16.63	2.11	0.71	2,125	17.38	1.92	2,340	16.44	2.07	2,188	16.11	2.14
	wave 3	6,079	16.45	2.24	0.71	1971	17.11	2.09	2,164	16.28	2.22	1944	15.98	2.26
	wave 4	4,600	16.28	2.21	0.70	1,214	16.85	2.11	1783	16.07	2.19	1,603	16.08	2.23

BIS, behavioural inhibition system scale; BAS, behavioural approach/activation system; BAS-d, drive subscale; BAS-f, fun-seeking subscale; BAS-r, reward responsiveness subscale; *n*, sample size; *SD*, standard deviation; and *α*, Cronbach’s alpha.

† Percent female.

‡ BAS-f not computed since items “*will often do things for no other reason than that they might be fun*” and “*I crave excitement and new sensations*” were missing in high proportion.

### Measures

#### Behavioural Inhibition and Behavioural Activation System Scales

The BIS/BAS scales (Carver and White, 1994) comprise 20 questions structured in a four-point Likert scale, with responses ranging from *very false for me* to *very true for me*. The additional four filler questions were not included in analyses. The BIS scale includes seven items assessing inhibitory behaviour and sensitivity to punishment, with items reflecting responses to potentially unpleasant scenarios. The BAS scale consists of three subscales. The drive subscale (BAS-d) includes four items reflecting the pursuit of goals; the fun-seeking subscale (BAS-f) includes four items reflecting willingness to approach rewarding situations; and the reward responsiveness subscale (BAS-r) includes five items reflecting positive responses to rewards.

#### Revised Eysenck Personality Questionnaire Short-Scale

The revised Eysenck personality questionnaire short-scale (EPQ-R; Eysenck et al., 1985), comprises 36 questions structured with dichotomous responses *yes* and *no* for three scales. The lie scale was not included in PATH. The extraversion scale includes 12 items addressing attitudes towards social interactions and external arousal; the neuroticism scale includes 12 items addressing attitudes towards feelings of worry and emotional insecurity; and the psychoticism scale includes 12 items addressing attitudes towards hostility and non-conformism.

#### Positive and Negative Affect Schedule

The positive and negative affect schedule (PANAS; Watson et al., 1988), comprises 20 questions in a five-point Likert scale with responses ranging from *very slightly or not at all* to *extremely*. The positive affect scale includes 10 descriptors assessing enthusiasm and alertness, and the negative affect scale includes 10 descriptors assessing distress and unpleasant states within the past 4 weeks.

#### Goldberg Anxiety and Depression Scales

The Goldberg anxiety and depression scales (GADS; Goldberg et al., 1988), comprise 18 questions structured with dichotomous responses *yes* and *no*. The anxiety scale includes nine questions assessing generalised anxiety disorder symptoms, and the depression scale includes nine questions assessing major depressive disorder symptoms within the past 4 weeks. The first four questions of each scale address core disorder symptoms, while the remaining questions address supplementary symptoms associated with physical disturbances. Scores of at least seven in the anxiety scale represent optimal screening values for generalised anxiety, and scores of at least five in the depression scale represent optimal screening values for major depressive disorder (Kiely and Butterworth, 2015).

### Statistical Analysis

R version 3.6.0 (R Core Team, 2019) for Windows was used for all statistical analyses. Exploratory factor analyses (EFAs) were conducted using the *psych* package version 1.8.12 (Revelle, 2018). Confirmatory factor analyses (CFAs) were conducted using the *lavaan* package version 0.6-3 (Rosseel, 2012; see Supplement for R code). A *p* ≤ 0.001 was considered significant for all analyses and our sample met size recommendations for factor analyses (Mundfrom et al., 2005; Li, 2016).

#### Exploratory Factor Analyses

Exploratory factor analyses were conducted to investigate the latent factor structure of the BIS/BAS scales. EFAs were conducted in a random subsample representing 60% of PATH wave 1 data, while the remaining 40% of the data was reserved for subsequent CFAs. There were no significant differences between EFA and CFA subsamples in terms of age, sex, education, or BIS/BAS scores (Supplementary Table 1). EFAs were estimated with weighted least squares (WLS) extraction owing to the distribution of the manifest item indicators, with an oblique Oblimin rotation to allow for possible correlations between factors, based on raw scores from a polychoric correlation matrix (Revelle, 2018). Several EFA models were investigated starting with a two-factor solution representing BIS and BAS as single general scales, and systematically increasing factor number until appropriate model fit was established. To ensure that the identified model represented the best fit for the data, a model with one extra factor was further investigated (Fabrigar and Wegener, 2012). The total number of factors examined was six.

#### Multiple-Groups Confirmatory Factor Analyses

Multiple-groups CFAs were used to assess measurement invariance (Jöreskog, 1971) of the EFA model. Age and sex invariance were assessed in the remaining 40% subsample of PATH wave 1 data, while longitudinal invariance was conducted with all waves. CFAs were conducted with diagonal WLS extraction allowing correlations between factors, based on raw scores from polychoric covariance matrices (Rosseel, 2012). This method has been shown to outperform robust maximum likelihood estimation when dealing with Likert-type scales with four response categories (Li, 2016). For model identification, the first variable of each latent factor was set to 1, latent factor variances were freely estimated, and intercepts were set to 0.

Measurement invariance was investigated using the method proposed by Wu and Estabrook (2016) for ordinal categorical data since previous methods, i.e., (Meredith, 1993), are based on continuous data. Briefly, this approach recognises the parameter constraints required for model identification as invariance constraints and imposes more stringent model parameters in a series of nested models. First, configural invariance is assessed with free estimation of thresholds and factor loadings within groups to ensure suitability of the model structure, with residual variances set to 1 and factor means set to 0 for all groups. Second, scalar invariance is assessed by fixing thresholds to be equal among groups, with residual variances set to 1 and factor means set to 0 in a reference group, and estimated in the remaining groups. Finally, metric invariance is investigated by fixing both thresholds and factor loadings to be equal among groups, with residual variances set to 1 and factor means set to 0 in the same reference group as scalar invariance, and estimated in the remaining groups (Supplementary Table 2). At each step, model fit is assessed. Compared to other methodologies (Meredith, 1993; Millsap and Yun-Tein, 2004), the testing order is reversed since it was demonstrated that metric invariance can only be tested if prior scalar invariance is established (Wu and Estabrook, 2016).

#### Model Fit

Several goodness of fit indicators were used to assess EFA and CFA models. The *χ*^2^ ratio test is sensitive to sample size (Bentler and Bonett, 1980; Windsor et al., 2012; Svetina et al., 2019) and small deviations in likelihood would reach statistical significance. Therefore, we included Tucker-Lewis index (*TLI*), comparative fit index (*CFI*), root mean square error of approximation (*RMSEA*), and Bayesian information criterion (*BIC*) as indicators. We considered a *TLI/CFI* ≥ 0.90, *RMSEA* ≤ 0.08, and the smallest *BIC* values as indicators of good fit. These threshold values are close to previous recommendations for normally distributed data (Hu and Bentler, 1999; Poythress et al., 2008; Fabrigar and Wegener, 2012).

#### Measurement Invariance

Measurement invariance is established when equality constraints yield appropriate model fit and there is a non-significant *χ*^2^ test between scalar and metric nested models (Wu and Estabrook, 2016; Svetina et al., 2019). However, the *χ*^2^ test is likely to reject invariant models with large number of groups and/or factors (Svetina and Rutkowski, 2017; Svetina et al., 2019). Therefore, we additionally considered a change in *RMSEA* (*ΔRMSEA*) ≤ 0.01 and change in *CFI* (*ΔCFI*) ≥ −0.002 between the two most restricted models as indicators of invariance (Svetina and Rutkowski, 2017).

#### Scale Correlations

Associations between BIS/BAS scales and EPQ-R, PANAS, and GADS were investigated in PATH wave 1 given the larger sample and data available. A CFA was conducted to obtain latent scores of the identified RST systems. Items 1 and 6 from the BIS scale were reverse coded to represent psychometrically meaningful associations with other scales.

Based on theoretical associations and previous literature, we expected the BIS scale to be positively associated to neuroticism, negative affect, and anxiety (Carver and White, 1994; Jorm et al., 1999; Gray and McNaughton, 2000; Johnson et al., 2003; Heym et al., 2008; Poythress et al., 2008; Jackson, 2009; Yu et al., 2011; Kramer and Rodriguez, 2018); and negatively associated to psychoticism (Jorm et al., 1999; Heym et al., 2008). We also expected all BAS subscales to be positively associated to extraversion and positive affect (Carver and White, 1994; Jorm et al., 1999; Gray and McNaughton, 2000; Smillie et al., 2006a; Heym et al., 2008; Beck et al., 2009; Kramer and Rodriguez, 2018), with BAS-f additionally positively associated to psychoticism (Jorm et al., 1999; Smillie et al., 2006a; Heym et al., 2008); and negatively associated to neuroticism and negative affect. We also expected a negative correlation between BAS and depression (Gray and McNaughton, 2000); however, a positive association between BIS and depression is likely given previous studies (Jorm et al., 1999; Johnson et al., 2003). Finally, we expected an FFFS scale to be positively associated to neuroticism and negative affect, and negatively associated to psychoticism based on previous literature (Heym et al., 2008; Kramer and Rodriguez, 2018).

## Results

### Latent Structure of the BIS/BAS Scales

We first investigated the latent factor structure of the BIS/BAS scales. The PATH wave 1 EFA subsample included 4,491 participants [mean age = 43.38 (SD = 16.40) years, 50.8% female]. All scales showed acceptable internal consistency based on pre-established items (*α* ≥ 0.7; Supplementary Table 1).

**Figure 1:**
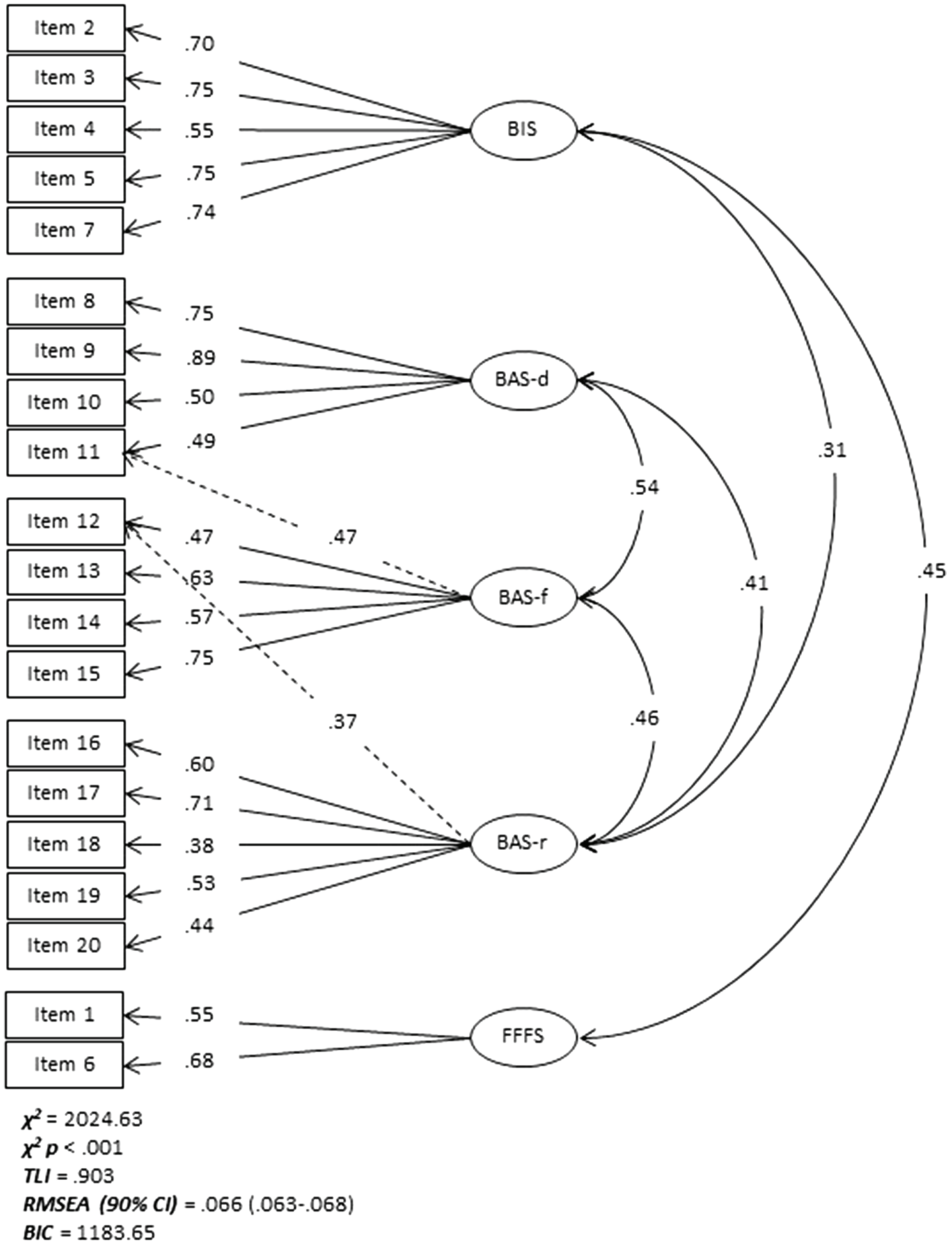
Behavioural inhibition system scale BIS/BAS/FFFS five-factor model. Loadings < 0.32 have been omitted. Dashed lines represent lower double loadings. BIS, behavioural inhibition system; BAS, behavioural approach/activation system; BAS-d, drive; BAS-f, fun-seeking; BAS-r, reward responsiveness; FFFS, fight-flight-freezing; *χ*^2^, Chi-square; *TLI*, Tucker Lewis index; *RMSEA*, root mean square error of approximation; *CI*, confidence interval; and *BIC*, bayesian information criterion.

A five-factor model best fitted the data, accounting for 52% of the variance. Addition of a sixth factor worsened fit indices, and therefore was not retained (Table 2; see Supplementary Figures 1, 2 for four- and six-factor models, respectively). The loading structure of the five-factor model showed BAS items divided into three factors representing each of the established BAS subscales, and BIS items divided into two factors with items 1 and 6 loading separately (Figure 1; Supplementary Table 3). Considering that items 1 (“*I have very few fears compared to my friends*”) and 6 (“*even when something bad is about to happen to me, I rarely experience fear or nervousness*”) are associated with fearful rather than anxious emotions, the fifth factor was identified as the FFFS.

**Table 2:** Exploratory factor analysis of the BIS/BAS scales.

Model	Model fit						
	*χ* ^2^	*χ* ^2^ *p*	*RMSR*	*TLI*	*RMSEA*	*RMSEA 90%CI*	*BIC*
Two-factor	6915.665	<0.001	0.06	0.775	0.100	0.098–0.102	5645.78
Three-factor	4488.771	<0.001	0.04	0.835	0.085	0.083–0.088	3370.26
Four-factor	2765.122	<0.001	0.03	0.885	**0.071**	**0.069–0.074**	1789.58
Five-factor	2024.633	<0.001	0.02	**0.903**	**0.066**	**0.063–0.068**	**1183.65**
Six-factor	1975.694	<0.001	0.02	0.888	**0.070**	**0.068–0.073**	1260.86

Exploratory factor analyses were conducted in a random sample representing 60% of wave 1 data. *χ*^2^, Chi-square value; *RMSR*, root mean square residual; *TLI*, tucker lewis index; *RMSEA*, root mean square error of approximation; *CI*, confidence interval; and *BIC*, bayesian information criterion. Bold font indicates appropriate fit indices.

Positive factor correlations (*r* ≥ 0.41) were found between the three BAS subscales, BIS and FFFS (*r* = 0.45), and BIS and BAS-r (*r* = 0.31).

### Measurement Invariance

#### Age and Sex Invariance

A series of increasingly restrictive nested models were conducted to establish invariance of the structure and assess the generalisability of our findings. The PATH wave 1 CFA subsample included 2,994 participants [mean age = 43.60 (*SD* = 16.14) years, 50.2% female; Supplementary Table 1]. Configural, scalar, and metric nested models showed appropriate fit indices for age and sex (Table 3; see Supplementary Tables 4, 5 for factor loadings and thresholds for fully invariant models, respectively). A comparison of the most restrictive models yielded significant *χ*^2^ tests as expected; however, *ΔRMSEA*s and *ΔCFI*s were below the established cut-offs indicating age and sex invariance.

**Table 3:** Measurement invariance models of the BIS/BAS/FFFS scales.

	Model fit							Invariance assessment				
	*χ* ^2^	*df*	*χ* ^2^ *p*	*CFI*	*TLI*	*RMSEA (90%CI)*	*RMSEA p*	*Δχ* ^2^	*Δdf*	*Δχ* ^2^ *p*	*ΔRMSEA*	*ΔCFI*
**Age**												
Configural	2,246.791	480	<0.001	**0.972**	**0.966**	**0.061 (0.059–0.064)**	<0.001					
Scalar	2,683.248	550	<0.001	**0.966**	**0.965**	**0.063 (0.061–0.065)**	<0.001					
Metric	2,825.096	580	<0.001	**0.964**	**0.965**	**0.063 (0.061–0.065)**	<0.001	77.178	30	<0.001	**0.000**	**0.002**
**Sex**												
Configural	2,189.642	320	<0.001	**0.972**	**0.966**	**0.063 (0.061–0.066)**	<0.001					
Scalar	2,331.972	355	<0.001	**0.970**	**0.968**	**0.062 (0.059–0.064)**	<0.001					
Metric	2,421.893	370	<0.001	**0.969**	**0.968**	**0.062 (0.059–0.064)**	<0.001	57.492	15	<0.001	**0.000**	**0.001**
**Longitudinal**												
Configural	41,773.392	2,770	<0.001	**0.976**	**0.972**	**0.043 (0.043–0.044)**	**1**					
Scalar	42,695.255	2,875	<0.001	**0.975**	**0.973**	**0.043 (0.043–0.043)**	**1**					
Metric	42,980.264	2,920	<0.001	**0.975**	**0.973**	**0.043 (0.043–0.043)**	**1**	211.840	45	<0.001	**0.000**	**0.001**

Age and sex invariance were conducted in a random sample representing 40% of wave 1 data, longitudinal invariance was conducted using all four waves. *χ*^2^, Chi-square value; *df*, degrees of freedom; *CFI*, comparative fit index; *TLI*, Tucker Lewis index; *RMSEA*, root mean square error of approximation; *CI*, confidence interval; and *Δ*, difference between scalar and metric models. Bold font indicates appropriate fit indices.

In the invariant models, the pattern of factor correlations was similar between cohorts but the magnitude varied by age and sex. Positive correlations between BIS and BAS-r were highest in old-age, with decreasing values as age decreased. Positive correlations between BIS and FFFS were highest in the young, with decreasing values as age increased. Positive correlations between BAS scales were highest in old-age and lowest in midlife; while negative correlations between BAS-d and BAS-f, and FFFS highest in the young, and lowest in midlife. Finally, positive correlations between BIS and BAS-r, and BIS and FFFS were highest in females; while positive correlations between BAS subscales, and negative correlations between BAS-d and BAS-r, and FFFS were highest in males (Supplementary Table 6).

#### Longitudinal Invariance

Configural, scalar, and metric nested models showed appropriate fit indices and were comparatively better than those for age and sex invariance (Table 3; see Supplementary Tables 4, 5 for factor loadings and thresholds for fully invariant models, respectively). Comparison of the most restrictive models yielded a significant *χ*^2^ test again, but *ΔRMSEA* and *ΔCFI* were below the established cut-offs indicating longitudinal invariance.

The pattern of moderate to high correlations in the longitudinal invariant model was similar between waves. There was low variability in the magnitude of these correlations when compared to the age and sex invariant models. Correlations between BAS-d and BAS-f showed the highest variability between waves (*range* = 0.64–0.72; Supplementary Table 7).

### Scale Correlations

The internal consistencies of extraversion, neuroticism, PANAS, and GADS scales were high (*α* ≥ 0.78); while the internal consistency of the psychoticism scale was poor (*α* = 0.48; Table 4). Most scales investigated showed significant sex differences (Mann-Whitney-Wilcoxon test, *p* < 0.001); however, correlations between scales did not differ substantially by sex and were thus investigated in the whole sample (Supplementary Figures 3, 4).

**Table 4:** Descriptive statistics of personality and mental health measures at wave 1.

		PATH wave 1				Females			Males			MWW test
		*n*	*Mean*	*SD*	*α*	*n*	*Mean*	*SD*	*n*	*Mean*	*SD*	*p*
EPQ-R	Extraversion	7,428	7.31	3.55	0.86	3,784	7.49	3.51	3,644	7.12	3.58	**<0.001**
	Neuroticism	7,434	4.05	3.28	0.84	3,785	4.61	3.29	3,649	3.47	3.17	**<0.001**
	Psychoticism	7,435	2.18	1.64	0.48	3,785	1.93	1.54	3,650	2.44	1.70	**<0.001**
PANAS	Positive affect	7,430	31.94	7.11	0.88	3,783	31.75	7.14	3,647	32.14	7.08	0.018
	Negative affect	7,433	16.38	6.44	0.90	3,784	16.98	6.88	3,649	15.77	5.88	**<0.001**
GADS	Anxiety	7,439	3.18	2.68	0.81	3,789	3.56	2.72	3,650	2.79	2.57	**<0.001**
	Depression	7,441	2.32	2.28	0.78	3,789	2.51	2.35	3,644	7.12	3.58	**<0.001**

EPQ-R, revised Eysenck personality questionnaire short scale; PANAS, positive and negative affect schedule; GADS, Goldberg anxiety and depression scales; *n*, sample size; *SD*, standard deviation; *α*, Cronbach’s alpha; and MWW, Mann-Whitney-Wilcoxon. Bold *p* values indicate significant differences.

**Figure 2:**
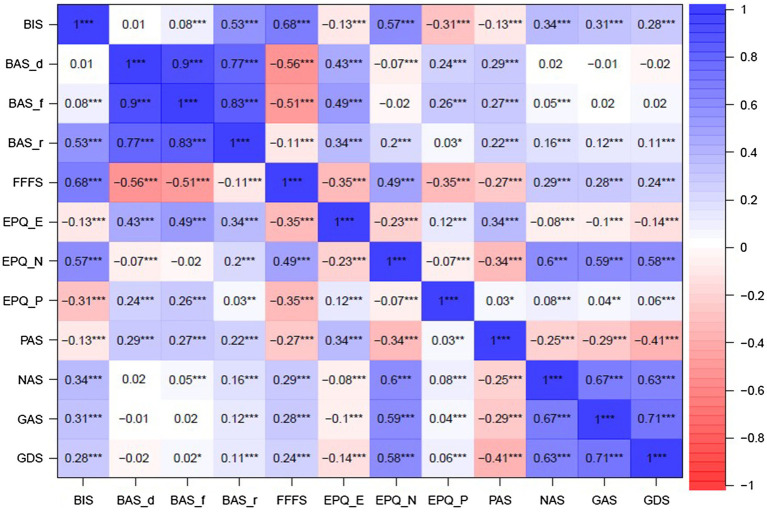
Associations between BIS/BAS/FFFS, EPQ-R, PANAS, and GADS at wave 1. Far right bar shows value of correlation. BIS, behavioural inhibition system; BAS, behavioural approach/activation system; BAS_d, drive; BAS_f, fun-seeking; BAS_r, reward responsiveness; FFFS, fight-flight-freezing; EPQ, Eysenck personality questionnaire; EPQ_E, extraversion; EPQ_N, neuroticism; EPQ_P, psychoticism; PAS, positive affect; NAS, negative affect; GAS, Goldberg anxiety; and GDS, Goldberg depression. ^*^p ≤ 0.05, ^**^p ≤ 0.001, and ^***^p ≤ 0.0001.

Significant positive correlations were found between BIS and neuroticism, negative affect, and anxiety and depression; between BAS subscales and extraversion, and positive affect; and between BAS-f and psychoticism. The magnitude of these correlations varied from *r* = 0.57 for BIS and neuroticism, to *r* = 0.22 for BAS-r and positive affect. A significant moderate negative correlation between BIS and psychoticism, and a significant moderate positive correlation between BAS-d and psychoticism were also found (Figure 2).

The pattern of correlations between FFFS and other scales largely resembled that of the BIS, except that positive correlations were greater with BIS and negative correlations were greater with FFFS. Significant positive correlations were found between FFFS and neuroticism, negative affect, and anxiety and depression; while significant negative correlations were found between FFFS and extraversion, psychoticism, and positive affect (Figure 2).

## Discussion

The present work contributes important data to behavioural and personality research. To the best of our knowledge, this is the first study to validate the BIS/BAS scales and investigate longitudinal invariance in a large community sample using contemporary methods that accurately analyse questionnaire data. Indeed, the methodology followed here was guided by methods and recommendations developed within the last 5 years (Li, 2016; Wu and Estabrook, 2016; Svetina and Rutkowski, 2017; Edossa et al., 2018; Svetina et al., 2019). The main finding of the study was that the BIS/BAS scales can effectively identify the three systems of the revised reinforcement sensitivity theory in a five-factor structure comprising the BIS, three BAS subscales, and the fear construct of the FFFS. Notably, this structure was found to be stable by age and sex, and, remarkably, within individuals over a period of 12 years.

### The BIS/BAS/FFFS Scales

The investigation of the factor structure of the BIS/BAS scales was primarily exploratory to identify the most parsimonious configuration of the data. We identified a BIS scale composed of five items instead of seven expected from the original BIS scale; three BAS subscales in line with the original BAS configuration; and a separate factor identified as the FFFS with items 1 and 6 from the original BIS scale. Consistent with this structure, items 1 and 6 have been previously shown to be distinct from the BIS scale (Johnson et al., 2003;Poythress et al., 2008; Beck et al., 2009) and measurement invariance demonstrated the stability of the BIS/BAS/FFFS scales.

This latter finding has direct implications for the FFFS scale, which includes the only two reverse-coded items and its separation from the BIS could result from a methodological artifact. We demonstrated using stringent analyses that a major component of the revised theory, the fear construct of the FFFS, is present, measureable and a robust construct to age, sex, and time stratification. Additionally, divergent associations between FFFS and BIS scales and other personality measures provide evidence of construct validity. Specifically, only FFFS items have been shown to associate with harm avoidance, while BIS items do not (Poythress et al., 2008); and only BIS items associate with BAS reward responsiveness, while FFFS items do not (Poythress et al., 2008; Beck et al., 2009). In this manner, the FFFS scale is linked to protective behaviours in line with the revised RST.

In contrast to this structure, a number of studies have proposed alternative configurations for the BIS/BAS scales. However, we consider that these results arise because of differences in analytical and methodological approaches. First with respect to analytical approach, item 4 from the original BIS scale (“*if I think something unpleasant is going to happen I usually get pretty* ‘*worked-up*’”) was additionally identified as part of the FFFS when the BIS scale was investigated in isolation (Heym et al., 2008). Considering that the scales represent RST systems that interact with one another, the investigation of latent constructs should follow a holistic approach, where the entirety of the questionnaire is assessed. Second with respect to methodology, previous studies that have supported the original four-factor structure (Heubeck et al., 1998; Jorm et al., 1999; Cooper et al., 2007), a three-factor structure without BAS fun-seeking scale (Pagliaccio et al., 2016), and a two-factor structure with a single BAS scale (Yu et al., 2011) have used CFA and/or invariance methodology designed for continuous data. Indeed, studies supporting a five-factor structure have used exploratory approaches (Johnson et al., 2003; Poythress et al., 2008) or CFA methodology designed for ordinal data (Beck et al., 2009) which are better suited for analysis.

### Personality and Mental Health Associations

Most associations between the BIS, BAS, and FFFS scales and personality and mental health measures followed theoretical expectations (Gray, 1970, 1982; Corr et al., 1995; Pickering and Gray, 1999; Gray and McNaughton, 2000; Corr, 2004), such as BIS and FFFS and negative affect, and BAS and extraversion. However, the reported associations between BIS and depression, FFFS and anxiety and depression, and BAS drive and psychoticism were unexpected theoretically (Figure 2).

The positive correlation between BIS and depression has been reported in literature (Jorm et al., 1999; Johnson et al., 2003; Yu et al., 2011), but it is unexpected since depression should reflect low BAS activation given anhedonic symptoms (Gray and McNaughton, 2000). However, we only found low correlations between BAS subscales and depression. Considering the high rates of comorbidity between anxiety and depression (Van Tol et al., 2010; World Health Organization, 2017; Espinoza Oyarce, et al., 2020), it is likely that the association between BIS and depression is attributable to anxiety as previously suggested (Bijttebier et al., 2009) and in line with the high correlation between anxiety and depression scales reported here. Our results show that in community-dwelling populations, anxiety and depression symptoms co-exist and this co-existence likely underlies the association between BIS and depression. This may not be a feature in other populations with clinically relevant depression symptoms and there is evidence of BAS hypoactivation in patients with ahedonic depressive symptoms, but not in patients with mixed anxiety and depression symptoms (Bijttebier et al., 2009). Future studies should investigate participants separately based on anxiety and depression symptoms to assess BIS and BAS sensitivities.

The unexpected positive correlations between FFFS and anxiety and depression may also result from underlying associations. Indeed, the FFFS is theoretically linked to neuroticism (Corr, 2004), which in turn is associated with both anxiety and depression. Our results show that these associations also extend to the fear component of the FFFS; however, similar to our discussion above, future research should investigate whether these associations hold in clinical populations, particularly those with panic and phobia symptoms. How the fear component of the FFFS relates to other aspects of human behaviour, such as health, diet, and life choices, remains the focus of future research.

Finally, the correlation between BAS drive subscale and psychoticism may require further investigation. This association has been reported in adults with the short-scale (Jorm et al., 1999) but not in undergraduate students with the full-scale (Smillie et al., 2006a; Heym et al., 2008). Age and demographics may explain these differences but the psychoticism scale likely plays a role. The full-scale showed higher internal consistencies and future investigations with the full-scale in adults would clarify the association between BAS and psychoticism.

### Implications for Personality and Behavioural Research

One of the major implications of our study is that the BIS, BAS, and FFFS scales are invariant constructs that can be used to study the RST in populations regardless of sex, age, and time between assessments. Furthermore, because the psychometric properties of these scales may reflect individual strengths and vulnerabilities to health and disease, the BIS, BAS, and FFFS scales could improve our understanding of the development of mental and physiological disorders and ultimately provide an avenue to prevent them.

For instance, individuals with high BIS scores would be predicted to be more sensitive to negative emotionality when an expected reward is not received, such as a salary raise, and more likely to have high anxiety and neuroticism. These conditions may translate in higher scores on anxiety and depression measures, and ultimately lead to clinical symptoms (Johnson et al., 2003; Poythress et al., 2008; Bijttebier et al., 2009). On the other hand, individuals with high BAS scores would be predicted to be more sensitive to positive emotionality when receiving a reward, such as a large meal after hard work, and more likely to have high extraversion and impulsivity; which may translate into higher risk of developing obesity and poorer cardiovascular health. Studies have shown that high BAS sensitivity is linked to purge/binge eating disorders (Beck et al., 2009; Bijttebier et al., 2009) and food craving (Franken and Muris, 2005), ultimately increasing obesity risk (Bijttebier et al., 2009) and development of associated metabolic diseases. There is, therefore, the need for more research aimed at identifying how BIS, BAS, and FFFS sensitivities relate to unhealthy behaviour and/or exposure to risk factors for physical and mental health. The scales here developed are suited for such investigation.

Another implication of our study is in the ongoing discussion of BAS dimensionality in behavioural research. Despite the theoretical, multidimensional nature of the BAS (Gray and McNaughton, 2000), there is currently no consensus on the dimensionality of psychometric measures of the BAS. New measures of the revised reinforcement sensitivity theory investigate BAS sensitivities using single scales, such as those proposed by Jackson (2009), Smederevac et al. (2014), and Reuter et al. (2015), and multiple scales, as proposed by Corr and Cooper (2016). Our results on the invariance of the BIS/BAS/FFFS scales agree with previous assessments that the BAS should be studied with separate scales given poor fit indices of unidimensional BAS scales (Krupić et al., 2016a). However, it is likely that the discussion of scale dimensionality will require a multimodal approach considering the neurobiological basis of the reinforcement sensitivity theory and reward circuitry, i.e., (Der-Avakian and Markou, 2011). Future studies could use the scales developed here to re-evaluate historical neuroimaging data, and neuroimaging studies comparing unidimensional and multidimensional scales will provide further insights on the psychometric dimensionality of the BAS to test the theoretical dimensionality of the system.

### Limitations and Future Directions

Despite the strengths of our study in terms of methodological rigor and longitudinal data, we must acknowledge some limitations. First, the FFFS scale addresses the central fear construct of the system, but specific responses to fight, flight, and freezing behaviour may not be fully captured. Similarly, the BIS scale may not fully assess conflict resolution. Future studies investigating the FFFS and BIS with newly developed measures, such as the RST personality questionnaire (Corr and Cooper, 2016) and the revised RST questionnaire (Reuter et al., 2015), should provide clearer insight of these traits. Complementary to this, future studies investigating associations between new measures and the BIS, BAS, and FFFS scales developed here would strengthen the link between historic and contemporary research based on the revised theory. Specifically, these studies could help corroborate or update previous associations between RST systems and behavioural, psychometric, and neuroimaging data that has been collected over two decades with the BIS/BAS scales. We have conducted such analyses for psychometric data and found correspondence between the BIS/BAS/FFFS scales here reported, newer measures with the revised RST questionnaire (Reuter et al., 2015), and personality measures (Submitted).

Second, we provide evidence of age, sex, and longitudinal invariance of the BIS, BAS, and FFFS scales; however, our sample included participants aged 20 years and older. Age invariance in younger cohorts remains a topic requiring further investigation and future studies should use the methodology presented here to investigate the five-factor structure of the BIS/BAS/FFFS scales in children and adolescents and assess whether other structures based on the old theory, i.e., (Muris et al., 2005), are better suited.

Further in regards to our sample, PATH participants have higher education level and socio-economic status compared to the national Australian average since the Canberra region is more socio-economically advantaged (Anstey et al., 2012). While we did not expect significant deviations on the measures investigated here, future studies with diverse socio-economic populations will provide further validation of the BIS, BAS, and FFFS scales.

Finally, psychometric associations with the psychoticism scale should be further investigated. The revised Eysenck personality questionnaire specifically addresses low internal consistency (Eysenck et al., 1985) yet low values are still reported, i.e., *α* = 0.24 (Tiwari et al., 2009). The multi-faceted nature of the scale, which includes lack of empathy, impulsivity and hostility, may underlie low consistencies (Eysenck et al., 1985).

## Conclusion

The Carver and White BIS/BAS scales produce robust and reliable measures that allow the investigation of the three systems of the revised reinforcement sensitivity theory. Future studies can use the framework proposed here to investigate the FFFS and BIS to assess behavioural responses linked to fear separate from those linked to anxiety. Furthermore, the validated BAS subscales will be instrumental in providing additional data to address the dimensionality of the BAS. Taken together, the BIS/BAS/FFFS scales will help build a conceptual bridge between historical and contemporary personality and behavioural research.

## Data Availability Statement

The datasets presented in this article are not readily available because PATH data is not publicly available. Although the PATH study governance arrangements and the commitments made to participants do not allow for unrestricted sharing of research data, we will be happy to make available the analysed data to external parties to verify our findings or for research purposes on a case by case basis after a formal request to, and approval from, the PATH Research Committee. Requests to access the datasets in this study should be directed to Prof Nicolas Cherbuin (nicolas.cherbuin@anu.edu.au). All other requests should be directed to info@pathstudy.org.au.

## Ethics Statement

The studies involving human participants were reviewed and approved by The Human Research Ethics Committee of The Australian National University. The participants provided their written informed consent to participate in this study.

## Author Contributions

DAEO contributed to the design of the study and statistical analyses, conducted all statistical analyses in R, and managed all aspects of manuscript preparation and submission. RB contributed to the design of the study, confirmed all statistical analyses in M*Plus*, provided methodological input and theoretical expertise, and contributed to writing and editing of the manuscript. PB and NC contributed to the design of the study and the statistical analyses, provided methodological input and theoretical expertise, and contributed to writing and editing of the manuscript. All authors contributed to the article and approved the submitted version.

## Funding

DAEO was supported by the Australian Government Research Training Program Scholarship. PATH data collection was supported by the National Health and Medical Research Council grants 973302, 179805, 418039, and 1002160 and Safe Work Australia. The funders had no role in study design, data analysis, data interpretation, or writing of the report. The corresponding author had full access to all the data in the study and all authors had final responsibility for the decision to submit for publication.

## Conflict of Interest

The authors declare that the research was conducted in the absence of any commercial or financial relationships that could be construed as a potential conflict of interest.

## Publisher’s Note

All claims expressed in this article are solely those of the authors and do not necessarily represent those of their affiliated organizations, or those of the publisher, the editors and the reviewers. Any product that may be evaluated in this article, or claim that may be made by its manufacturer, is not guaranteed or endorsed by the publisher.

## Abbreviations

RST: Reinforcement sensitivity theory
BIS: Behavioural inhibition system
BAS: Behavioural approach/activation system
FFS: Fight/flight system
FFFS: Fight-flight-freezing system
EPQ-R: Revised Eysenck personality questionnaire short-scale
PANAS: Positive and negative affect schedule
GADS: Goldberg anxiety and depression scales
