# Supplementary material for: Bridging Classical and Revised Reinforcement Sensitivity Theory Research: A Longitudinal Analysis of a Large Population Study
Source: Front Psychol. 2021 Dec 20;12:737117. doi: 10.3389/fpsyg.2021.737117 (PMC8771200; doi:10.3389/fpsyg.2021.737117)
Supplement: Supplementary file 1 [file Data_Sheet_1.docx]

Supplementary Material

# Supplementary Data

**R code for latent structure of the BIS/BAS scales**

**Exploratory factor analysis using *psych***

Missing data management: corrected for continuity (Revelle, 2018). Similar results found with pairwise deletion and no continuity correction (data not shown).

# w1_bisbasR dataset includes reversed-coded items 1 and 6 from BIS scale

#

# Run EFA with 2-factor solution:

#

EFA2_w1_bisbasR <- fa(w1_bisbasR, nfactors = 2, rotate = "oblimin", fm = "wls", cor = "poly")

#

# Show model fit:

#

EFA2_w1_bisbasR

#

# 3-factor solution:

#

EFA3_w1_bisbasR <- fa(w1_bisbasR, nfactors = 3, rotate = "oblimin", fm = "wls", cor = "poly")

#

# Show model fit:

#

EFA3_w1_bisbasR

#

# 4-factor solution:

#

EFA4_w1_bisbasR <- fa(w1_bisbasR, nfactors = 4, rotate = "oblimin", fm = "wls", cor = "poly")

#

# Show model fit:

#

EFA4_w1_bisbasR

#

# 5-factor solution:

#

EFA5_w1_bisbasR <- fa(w1_bisbasR, nfactors = 5, rotate = "oblimin", fm = "wls", cor = "poly")

#

# Show model fit:

#

EFA5_w1_bisbasR

#

# Inspect factor loadings using 0.3 as cut-off:

#

w1_bisbas5_loadR <- print(EFA5_w1_bisbasR$loadings, cutoff = 0.3)

#

# 6-factor solution:

#

EFA6_w1_bisbasR <- fa(w1_bisbasR, nfactors = 6, rotate = "oblimin", fm = "wls", cor = "poly")

#

# Show model fit:

#

EFA6_w1_bisbasR

#

# Inspect factor loadings using 0.3 as cut-off:

#

w1_bisbas6_load <- print(EFA6_w1_bisbas$loadings, cutoff = 0.3)

**R code for age, sex, and longitudinal invariance**

**Multiple-groups confirmatory factor analysis using *lavaan***

Missing data management: none, pairwise deletion (Rosseel, 2012).

**Age invariance**

# Model definition:

#

bb_model_R <- '# Latent factors:

BIS =~ bisbas8_w1 + bisbas13_w1 + bisbas16_w1 + bisbas19_w1

+ bisbas24_w1

BAS_d =~ bisbas3_w1 + bisbas9_w1 + bisbas12_w1 + bisbas21_w1

BAS_f =~ bisbas5_w1 + bisbas10_w1 + bisbas15_w1 + bisbas20_w1

BAS_r =~ bisbas4_w1 + bisbas7_w1 + bisbas14_w1 + bisbas18_w1 + bisbas23_w1

FFFS =~ bisbas22_w1R + bisbas2_w1R

# Factor correlations:

BIS ~~ BAS_d + BAS_f + BAS_r + FFFS

BAS_d ~~ BAS_f + BAS_r + FFFS

BAS_f ~~ BAS_r + FFFS

BAS_r ~~ FFFS'

#

# Measurement invariance model 1: configural invariance

#

age.fit_bb_model1 <- cfa(bb_model_R, data = CFA_path, ordered = c("bisbas8_w1", "bisbas13_w1", "bisbas16_w1", "bisbas19_w1", "bisbas24_w1", "bisbas3_w1", "bisbas9_w1", "bisbas12_w1", "bisbas21_w1", "bisbas5_w1", "bisbas10_w1", "bisbas15_w1", "bisbas20_w1", "bisbas4_w1", "bisbas7_w1", "bisbas14_w1", "bisbas18_w1", "bisbas23_w1", "bisbas22_w1R", "bisbas2_w1R"), parameterization = "theta", group = "age_grp", mimic = "Mplus")

#

# Show fit measures:

#

summary(age.fit_bb_model1, fit.measures = TRUE)

#

# Measurement invariance model 2: threshold invariance

#

age.fit_bb_model2 <- cfa(bb_model_R, data = CFA_path, ordered = c("bisbas8_w1", "bisbas13_w1", "bisbas16_w1", "bisbas19_w1", "bisbas24_w1", "bisbas3_w1", "bisbas9_w1", "bisbas12_w1", "bisbas21_w1", "bisbas5_w1", "bisbas10_w1", "bisbas15_w1", "bisbas20_w1", "bisbas4_w1", "bisbas7_w1", "bisbas14_w1", "bisbas18_w1", "bisbas23_w1", "bisbas22_w1R", "bisbas2_w1R"), parameterization = "theta", group = "age_grp", group.equal = c("thresholds"), mimic = "Mplus")

#

# Show fit measures:

#

summary(age.fit_bb_model2, fit.measures = TRUE)

#

# Measurement invariance model 3: loading invariance

#

age.fit_bb_model3 <- cfa(bb_model_R, data = CFA_path, ordered = c("bisbas8_w1", "bisbas13_w1", "bisbas16_w1", "bisbas19_w1", "bisbas24_w1", "bisbas3_w1", "bisbas9_w1", "bisbas12_w1", "bisbas21_w1", "bisbas5_w1", "bisbas10_w1", "bisbas15_w1", "bisbas20_w1", "bisbas4_w1", "bisbas7_w1", "bisbas14_w1", "bisbas18_w1", "bisbas23_w1", "bisbas22_w1R", "bisbas2_w1R"), parameterization = "theta", group = "age_grp", group.equal = c("thresholds", "loadings"), mimic = "Mplus")

#

# Show fit measures:

#

summary(age.fit_bb_model3, fit.measures = TRUE)

**Sex Invariance**

# Model definition:

#

bb_model_R <- '# Latent factors:

BIS =~ bisbas8_w1 + bisbas13_w1 + bisbas16_w1 + bisbas19_w1

+ bisbas24_w1

BAS_d =~ bisbas3_w1 + bisbas9_w1 + bisbas12_w1 + bisbas21_w1

BAS_f =~ bisbas5_w1 + bisbas10_w1 + bisbas15_w1

+ bisbas20_w1

BAS_r =~ bisbas4_w1 + bisbas7_w1 + bisbas14_w1 + bisbas18_w1

+ bisbas23_w1

FFFS =~ bisbas22_w1R + bisbas2_w1R

# Factor correlations:

BIS ~~ BAS_d + BAS_f + BAS_r + FFFS

BAS_d ~~ BAS_f + BAS_r + FFFS

BAS_f ~~ BAS_r + FFFS

BAS_r ~~ FFFS'

#

# Measurement invariance model 1: configural invariance

#

sex.fit_bb_model1 <- cfa(bb_model_R, data = CFA_path, ordered = c("bisbas8_w1", "bisbas13_w1", "bisbas16_w1", "bisbas19_w1", "bisbas24_w1", "bisbas3_w1", "bisbas9_w1", "bisbas12_w1", "bisbas21_w1", "bisbas5_w1", "bisbas10_w1", "bisbas15_w1", "bisbas20_w1", "bisbas4_w1", "bisbas7_w1", "bisbas14_w1", "bisbas18_w1", "bisbas23_w1", "bisbas22_w1R", "bisbas2_w1R"), parameterization = "theta", group = "gender", mimic = "Mplus")

#

# Show fit measures:

#

summary(sex.fit_bb_model1, fit.measures = TRUE)

#

#Measurement invariance model 2: threshold invariance

#

sex.fit_bb_model2 <- cfa(bb_model_R, data = CFA_path, ordered = c("bisbas8_w1", "bisbas13_w1", "bisbas16_w1", "bisbas19_w1", "bisbas24_w1", "bisbas3_w1", "bisbas9_w1", "bisbas12_w1", "bisbas21_w1", "bisbas5_w1", "bisbas10_w1", "bisbas15_w1", "bisbas20_w1", "bisbas4_w1", "bisbas7_w1", "bisbas14_w1", "bisbas18_w1", "bisbas23_w1", "bisbas22_w1R", "bisbas2_w1R"), parameterization = "theta", group = "gender", group.equal = c("thresholds"), mimic = "Mplus")

#

# Show fit measures:

#

summary(sex.fit_bb_model2, fit.measures = TRUE)

#

# Measurement invariance model 3: loading invariance

#

sex.fit_bb_model3 <- cfa(bb_model_R, data = CFA_path, ordered = c("bisbas8_w1", "bisbas13_w1", "bisbas16_w1", "bisbas19_w1", "bisbas24_w1", "bisbas3_w1", "bisbas9_w1", "bisbas12_w1", "bisbas21_w1", "bisbas5_w1", "bisbas10_w1", "bisbas15_w1", "bisbas20_w1", "bisbas4_w1", "bisbas7_w1", "bisbas14_w1", "bisbas18_w1", "bisbas23_w1", "bisbas22_w1R", "bisbas2_w1R"), parameterization = "theta", group = "gender", group.equal = c("thresholds", "loadings"), mimic = "Mplus")

#

# Show fit measures:

#

summary(sex.fit_bb_model3, fit.measures = TRUE)

**Longitudinal Invariance**

**Configural Invariance**

# Model definition:

#

Emi.bb_model1 <- '# Latent factors

BIS1 =~ bisbas8_w1 + bisbas13_w1 + bisbas16_w1

+ bisbas19_w1+ bisbas24_w1

BIS2 =~ bisbas8_w2 + bisbas13_w2 + bisbas16_w2

+ bisbas19_w2+ bisbas24_w2

BIS3 =~ bisbas8_w3 + bisbas13_w3 + bisbas16_w3

+ bisbas19_w3+ bisbas24_w3

BIS4 =~ bisbas8_w4 + bisbas13_w4 + bisbas16_w4

+ bisbas19_w4+ bisbas24_w4

BAS_d1 =~ bisbas3_w1 + bisbas9_w1 + bisbas12_w1

+ bisbas21_w1

BAS_d2 =~ bisbas3_w2 + bisbas9_w2 + bisbas12_w2

+ bisbas21_w2

BAS_d3 =~ bisbas3_w3 + bisbas9_w3 + bisbas12_w3

+ bisbas21_w3

BAS_d4 =~ bisbas3_w4 + bisbas9_w4 + bisbas12_w4

+ bisbas21_w4

BAS_f1 =~ bisbas5_w1 + bisbas10_w1 + bisbas15_w1

+ bisbas20_w1

BAS_f2 =~ bisbas5_w2 + bisbas10_w2 + bisbas15_w2

+ bisbas20_w2

BAS_f3 =~ bisbas5_w3 + bisbas10_w3 + bisbas15_w3

+ bisbas20_w3

BAS_f4 =~ bisbas5_w4 + bisbas10_w4 + bisbas15_w4

+ bisbas20_w4

BAS_r1 =~ bisbas4_w1 + bisbas7_w1 + bisbas14_w1

+ bisbas18_w1 + bisbas23_w1

BAS_r2 =~ bisbas4_w2 + bisbas7_w2 + bisbas14_w2

+ bisbas18_w2 + bisbas23_w2

BAS_r3 =~ bisbas4_w3 + bisbas7_w3 + bisbas14_w3

+ bisbas18_w3 + bisbas23_w3

BAS_r4 =~ bisbas4_w4 + bisbas7_w4 + bisbas14_w4

+ bisbas18_w4 + bisbas23_w4

FFFS1 =~ bisbas22_w1R + bisbas2_w1R

FFFS2 =~ bisbas22_w2R + bisbas2_w2R

FFFS3 =~ bisbas22_w3R + bisbas2_w3R

FFFS4 =~ bisbas22_w4R + bisbas2_w4R

# Latent factor covariances across waves

BIS1 ~~ BIS2 + BIS3 + BIS4

BIS2 ~~ BIS3 + BIS4

BIS3 ~~ BIS4

BAS_d1 ~~ BAS_d2 + BAS_d3 + BAS_d4

BAS_d2 ~~ BAS_d3 + BAS_d4

BAS_d3 ~~ BAS_d4

BAS_f1 ~~ BAS_f2 + BAS_f3 + BAS_f4

BAS_f2 ~~ BAS_f3 + BAS_f4

BAS_f3 ~~ BAS_f4

BAS_r1 ~~ BAS_r2 + BAS_r3 + BAS_r4

BAS_r2 ~~ BAS_r3 + BAS_r4

BAS_r3 ~~ BAS_r4

FFFS1 ~~ FFFS2 + FFFS3 + FFFS4

FFFS2 ~~ FFFS3 + FFFS4

FFFS3 ~~ FFFS4

# Latent factor covariances within waves

# Wave 1

BIS1 ~~ BAS_d1 + BAS_f1 + BAS_r1 + FFFS1

BAS_d1 ~~ BAS_f1 + BAS_r1 + FFFS1

BAS_f1 ~~ BAS_r1 + FFFS1

BAS_r1 ~~ FFFS1

# Wave 2

BIS2 ~~ BAS_d2 + BAS_f2 + BAS_r2 + FFFS2

BAS_d2 ~~ BAS_f2 + BAS_r2 + FFFS2

BAS_f2 ~~ BAS_r2 + FFFS2

BAS_r2 ~~ FFFS2

# Wave 3

BIS3 ~~ BAS_d3 + BAS_f3 + BAS_r3 + FFFS3

BAS_d3 ~~ BAS_f3 + BAS_r3 + FFFS3

BAS_f3 ~~ BAS_r3 + FFFS3

BAS_r3 ~~ FFFS3

# Wave 4

BIS4 ~~ BAS_d4 + BAS_f4 + BAS_r4 + FFFS4

BAS_d4 ~~ BAS_f4 + BAS_r4 + FFFS4

BAS_f4 ~~ BAS_r4 + FFFS4

BAS_r4 ~~ FFFS4

# Latent factor means

# Fix mean to zero at all waves

BIS1 ~ 0*1

BIS2 ~ 0*1

BIS3 ~ 0*1

BIS4 ~ 0*1

BAS_d1 ~ 0*1

BAS_d2 ~ 0*1

BAS_d3 ~ 0*1

BAS_d4 ~ 0*1

BAS_f1 ~ 0*1

BAS_f2 ~ 0*1

BAS_f3 ~ 0*1

BAS_f4 ~ 0*1

BAS_r1 ~ 0*1

BAS_r2 ~ 0*1

BAS_r3 ~ 0*1

BAS_r4 ~ 0*1

FFFS1 ~ 0*1

FFFS2 ~ 0*1

FFFS3 ~ 0*1

FFFS4 ~ 0*1

# All intercepts are set to zero as part of model definition

by lavaan

# All measured variable variances are set to one as part of

model definition by lavaan

# Unique factor covariances across waves

# BIS

bisbas8_w1 ~~ bisbas8_w2 + bisbas8_w3 + bisbas8_w4

bisbas8_w2 ~~ bisbas8_w3 + bisbas8_w4

bisbas8_w3 ~~ bisbas8_w4

bisbas13_w1 ~~ bisbas13_w2 + bisbas13_w3 + bisbas13_w4

bisbas13_w2 ~~ bisbas13_w3 + bisbas13_w4

bisbas13_w3 ~~ bisbas13_w4

bisbas16_w1 ~~ bisbas16_w2 + bisbas16_w3 + bisbas16_w4

bisbas16_w2 ~~ bisbas16_w3 + bisbas16_w4

bisbas16_w3 ~~ bisbas16_w4

bisbas19_w1 ~~ bisbas19_w2 + bisbas19_w3 + bisbas19_w4

bisbas19_w2 ~~ bisbas19_w3 + bisbas19_w4

bisbas19_w3 ~~ bisbas19_w4

bisbas24_w1 ~~ bisbas24_w2 + bisbas24_w3 + bisbas24_w4

bisbas24_w2 ~~ bisbas24_w3 + bisbas24_w4

bisbas24_w3 ~~ bisbas24_w4

# BAS_d

bisbas3_w1 ~~ bisbas3_w2 + bisbas3_w3 + bisbas3_w4

bisbas3_w2 ~~ bisbas3_w3 + bisbas3_w4

bisbas3_w3 ~~ bisbas3_w4

bisbas9_w1 ~~ bisbas9_w2 + bisbas9_w3 + bisbas9_w4

bisbas9_w2 ~~ bisbas9_w3 + bisbas9_w4

bisbas9_w3 ~~ bisbas9_w4

bisbas12_w1 ~~ bisbas12_w2 + bisbas12_w3 + bisbas12_w4

bisbas12_w2 ~~ bisbas12_w3 + bisbas12_w4

bisbas12_w3 ~~ bisbas12_w4

bisbas21_w1 ~~ bisbas21_w2 + bisbas21_w3 + bisbas21_w4

bisbas21_w2 ~~ bisbas21_w3 + bisbas21_w4

bisbas21_w3 ~~ bisbas21_w4

# BAS_f

bisbas5_w1 ~~ bisbas5_w2 + bisbas5_w3 + bisbas5_w4

bisbas5_w2 ~~ bisbas5_w3 + bisbas5_w4

bisbas5_w3 ~~ bisbas5_w4

bisbas10_w1 ~~ bisbas10_w2 + bisbas10_w3 + bisbas10_w4

bisbas10_w2 ~~ bisbas10_w3 + bisbas10_w4

bisbas10_w3 ~~ bisbas10_w4

bisbas15_w1 ~~ bisbas15_w2 + bisbas15_w3 + bisbas15_w4

bisbas15_w2 ~~ bisbas15_w3 + bisbas15_w4

bisbas15_w3 ~~ bisbas15_w4

bisbas20_w1 ~~ bisbas20_w2 + bisbas20_w3 + bisbas20_w4

bisbas20_w2 ~~ bisbas20_w3 + bisbas20_w4

bisbas20_w3 ~~ bisbas20_w4

# BAS_r

bisbas4_w1 ~~ bisbas4_w2 + bisbas4_w3 + bisbas4_w4

bisbas4_w2 ~~ bisbas4_w3 + bisbas4_w4

bisbas4_w3 ~~ bisbas4_w4

bisbas7_w1 ~~ bisbas7_w2 + bisbas7_w3 + bisbas7_w4

bisbas7_w2 ~~ bisbas7_w3 + bisbas7_w4

bisbas7_w3 ~~ bisbas7_w4

bisbas14_w1 ~~ bisbas14_w2 + bisbas14_w3 + bisbas14_w4

bisbas14_w2 ~~ bisbas14_w3 + bisbas14_w4

bisbas14_w3 ~~ bisbas14_w4

bisbas18_w1 ~~ bisbas18_w2 + bisbas18_w3 + bisbas18_w4

bisbas18_w2 ~~ bisbas18_w3 + bisbas18_w4

bisbas18_w3 ~~ bisbas18_w4

bisbas23_w1 ~~ bisbas23_w2 + bisbas23_w3 + bisbas23_w4

bisbas23_w2 ~~ bisbas23_w3 + bisbas23_w4

bisbas23_w3 ~~ bisbas23_w4

# FFFS

bisbas22_w1R ~~ bisbas22_w2R + bisbas22_w3R + bisbas22_w4R

bisbas22_w2R ~~ bisbas22_w3R + bisbas22_w4R

bisbas22_w3R ~~ bisbas22_w4R

bisbas2_w1R ~~ bisbas2_w2R + bisbas2_w3R + bisbas2_w4R

bisbas2_w2R ~~ bisbas2_w3R + bisbas2_w4R

bisbas2_w3R ~~ bisbas2_w4R'

#

# Fit model:

#

fit_Emi.bb_model1 <- cfa(Emi.bb_model1, data = PATH, ordered = c("bisbas8_w1", "bisbas8_w2", "bisbas8_w3", "bisbas8_w4", "bisbas13_w1", "bisbas13_w2", "bisbas13_w3", "bisbas13_w4", "bisbas16_w1", "bisbas16_w2", "bisbas16_w3", "bisbas16_w4", "bisbas19_w1", "bisbas19_w2", "bisbas19_w3", "bisbas19_w4", "bisbas24_w1", "bisbas24_w2", "bisbas24_w3", "bisbas24_w4", "bisbas3_w1", "bisbas3_w2", "bisbas3_w3", "bisbas3_w4", "bisbas9_w1", "bisbas9_w2", "bisbas9_w3", "bisbas9_w4", "bisbas12_w1", "bisbas12_w2", "bisbas12_w3", "bisbas12_w4", "bisbas21_w1", "bisbas21_w2", "bisbas21_w3", "bisbas21_w4", "bisbas5_w1", "bisbas5_w2", "bisbas5_w3", "bisbas5_w4", "bisbas10_w1", "bisbas10_w2", "bisbas10_w3", "bisbas10_w4", "bisbas15_w1", "bisbas15_w2", "bisbas15_w3", "bisbas15_w4", "bisbas20_w1", "bisbas20_w2", "bisbas20_w3", "bisbas20_w4", "bisbas4_w1", "bisbas4_w2", "bisbas4_w3", "bisbas4_w4", "bisbas7_w1", "bisbas7_w2", "bisbas7_w3", "bisbas7_w4", "bisbas14_w1", "bisbas14_w2", "bisbas14_w3", "bisbas14_w4", "bisbas18_w1", "bisbas18_w2", "bisbas18_w3", "bisbas18_w4", "bisbas23_w1", "bisbas23_w2", "bisbas23_w3", "bisbas23_w4", "bisbas22_w1R", "bisbas22_w2R", "bisbas22_w3R", "bisbas22_w4R", "bisbas2_w1R", "bisbas2_w2R", "bisbas2_w3R", "bisbas2_w4R"), parameterization = "theta", missing = "pairwise", mimic = "Mplus")

#

# Show fit measures:

#

summary(fit_Emi.bb_model1, fit.measures = TRUE)

**Threshold Invariance**

# Model definition:

#

Emi.bb_model2 <- '# Latent factors

BIS1 =~ bisbas8_w1 + bisbas13_w1 + bisbas16_w1

+ bisbas19_w1+ bisbas24_w1

BIS2 =~ bisbas8_w2 + bisbas13_w2 + bisbas16_w2

+ bisbas19_w2+ bisbas24_w2

BIS3 =~ bisbas8_w3 + bisbas13_w3 + bisbas16_w3

+ bisbas19_w3+ bisbas24_w3

BIS4 =~ bisbas8_w4 + bisbas13_w4 + bisbas16_w4

+ bisbas19_w4+ bisbas24_w4

BAS_d1 =~ bisbas3_w1 + bisbas9_w1 + bisbas12_w1

+ bisbas21_w1

BAS_d2 =~ bisbas3_w2 + bisbas9_w2 + bisbas12_w2

+ bisbas21_w2

BAS_d3 =~ bisbas3_w3 + bisbas9_w3 + bisbas12_w3

+ bisbas21_w3

BAS_d4 =~ bisbas3_w4 + bisbas9_w4 + bisbas12_w4

+ bisbas21_w4

BAS_f1 =~ bisbas5_w1 + bisbas10_w1 + bisbas15_w1

+ bisbas20_w1

BAS_f2 =~ bisbas5_w2 + bisbas10_w2 + bisbas15_w2

+ bisbas20_w2

BAS_f3 =~ bisbas5_w3 + bisbas10_w3 + bisbas15_w3

+ bisbas20_w3

BAS_f4 =~ bisbas5_w4 + bisbas10_w4 + bisbas15_w4

+ bisbas20_w4

BAS_r1 =~ bisbas4_w1 + bisbas7_w1 + bisbas14_w1

+ bisbas18_w1 + bisbas23_w1

BAS_r2 =~ bisbas4_w2 + bisbas7_w2 + bisbas14_w2

+ bisbas18_w2 + bisbas23_w2

BAS_r3 =~ bisbas4_w3 + bisbas7_w3 + bisbas14_w3

+ bisbas18_w3 + bisbas23_w3

BAS_r4 =~ bisbas4_w4 + bisbas7_w4 + bisbas14_w4

+ bisbas18_w4 + bisbas23_w4

FFFS1 =~ bisbas22_w1R + bisbas2_w1R

FFFS2 =~ bisbas22_w2R + bisbas2_w2R

FFFS3 =~ bisbas22_w3R + bisbas2_w3R

FFFS4 =~ bisbas22_w4R + bisbas2_w4R

# Latent factor covariances across waves

BIS1 ~~ BIS2 + BIS3 + BIS4

BIS2 ~~ BIS3 + BIS4

BIS3 ~~ BIS4

BAS_d1 ~~ BAS_d2 + BAS_d3 + BAS_d4

BAS_d2 ~~ BAS_d3 + BAS_d4

BAS_d3 ~~ BAS_d4

BAS_f1 ~~ BAS_f2 + BAS_f3 + BAS_f4

BAS_f2 ~~ BAS_f3 + BAS_f4

BAS_f3 ~~ BAS_f4

BAS_r1 ~~ BAS_r2 + BAS_r3 + BAS_r4

BAS_r2 ~~ BAS_r3 + BAS_r4

BAS_r3 ~~ BAS_r4

FFFS1 ~~ FFFS2 + FFFS3 + FFFS4

FFFS2 ~~ FFFS3 + FFFS4

FFFS3 ~~ FFFS4

# Latent factor covariances within waves

# Wave 1

BIS1 ~~ BAS_d1 + BAS_f1 + BAS_r1 + FFFS1

BAS_d1 ~~ BAS_f1 + BAS_r1 + FFFS1

BAS_f1 ~~ BAS_r1 + FFFS1

BAS_r1 ~~ FFFS1

# Wave 2

BIS2 ~~ BAS_d2 + BAS_f2 + BAS_r2 + FFFS2

BAS_d2 ~~ BAS_f2 + BAS_r2 + FFFS2

BAS_f2 ~~ BAS_r2 + FFFS2

BAS_r2 ~~ FFFS2

# Wave 3

BIS3 ~~ BAS_d3 + BAS_f3 + BAS_r3 + FFFS3

BAS_d3 ~~ BAS_f3 + BAS_r3 + FFFS3

BAS_f3 ~~ BAS_r3 + FFFS3

BAS_r3 ~~ FFFS3

# Wave 4

BIS4 ~~ BAS_d4 + BAS_f4 + BAS_r4 + FFFS4

BAS_d4 ~~ BAS_f4 + BAS_r4 + FFFS4

BAS_f4 ~~ BAS_r4 + FFFS4

BAS_r4 ~~ FFFS4

# Latent factor means

# Fix mean to zero only at wave 1, free at all other waves

BIS1 ~ 0*1

BIS2 ~ NA*1

BIS3 ~ NA*1

BIS4 ~ NA*1

BAS_d1 ~ 0*1

BAS_d2 ~ NA*1

BAS_d3 ~ NA*1

BAS_d4 ~ NA*1

BAS_f1 ~ 0*1

BAS_f2 ~ NA*1

BAS_f3 ~ NA*1

BAS_f4 ~ NA*1

BAS_r1 ~ 0*1

BAS_r2 ~ NA*1

BAS_r3 ~ NA*1

BAS_r4 ~ NA*1

FFFS1 ~ 0*1

FFFS2 ~ NA*1

FFFS3 ~ NA*1

FFFS4 ~ NA*1

# All intercepts are set to zero as part of model definition

by lavaan

# Fix thresholds to be identical between waves

# BIS

bisbas8_w1 | A1*t1 + A2*t2 + A3*t3

bisbas13_w1 | B1*t1 + B2*t2 + B3*t3

bisbas16_w1 | C1*t1 + C2*t2 + C3*t3

bisbas19_w1 | D1*t1 + D2*t2 + D3*t3

bisbas24_w1 | E1*t1 + E2*t2 + E3*t3

bisbas8_w2 | A1*t1 + A2*t2 + A3*t3

bisbas13_w2 | B1*t1 + B2*t2 + B3*t3

bisbas16_w2 | C1*t1 + C2*t2 + C3*t3

bisbas19_w2 | D1*t1 + D2*t2 + D3*t3

bisbas24_w2 | E1*t1 + E2*t2 + E3*t3

bisbas8_w3 | A1*t1 + A2*t2 + A3*t3

bisbas13_w3 | B1*t1 + B2*t2 + B3*t3

bisbas16_w3 | C1*t1 + C2*t2 + C3*t3

bisbas19_w3 | D1*t1 + D2*t2 + D3*t3

bisbas24_w3 | E1*t1 + E2*t2 + E3*t3

bisbas8_w4 | A1*t1 + A2*t2 + A3*t3

bisbas13_w4 | B1*t1 + B2*t2 + B3*t3

bisbas16_w4 | C1*t1 + C2*t2 + C3*t3

bisbas19_w4 | D1*t1 + D2*t2 + D3*t3

bisbas24_w4 | E1*t1 + E2*t2 + E3*t3

# BAS_d

bisbas3_w1 | F1*t1 + F2*t2 + F3*t3

bisbas9_w1 | G1*t1 + G2*t2 + G3*t3

bisbas12_w1 | H1*t1 + H2*t2 + H3*t3

bisbas21_w1 | I1*t1 + I2*t2 + I3*t3

bisbas3_w2 | F1*t1 + F2*t2 + F3*t3

bisbas9_w2 | G1*t1 + G2*t2 + G3*t3

bisbas12_w2 | H1*t1 + H2*t2 + H3*t3

bisbas21_w2 | I1*t1 + I2*t2 + I3*t3

bisbas3_w3 | F1*t1 + F2*t2 + F3*t3

bisbas9_w3 | G1*t1 + G2*t2 + G3*t3

bisbas12_w3 | H1*t1 + H2*t2 + H3*t3

bisbas21_w3 | I1*t1 + I2*t2 + I3*t3

bisbas3_w4 | F1*t1 + F2*t2 + F3*t3

bisbas9_w4 | G1*t1 + G2*t2 + G3*t3

bisbas12_w4 | H1*t1 + H2*t2 + H3*t3

bisbas21_w4 | I1*t1 + I2*t2 + I3*t3

# BAS_f

bisbas5_w1 | J1*t1 + J2*t2 + J3*t3

bisbas10_w1 | K1*t1 + K2*t2 + K3*t3

bisbas15_w1 | L1*t1 + L2*t2 + L3*t3

bisbas20_w1 | M1*t1 + M2*t2 + M3*t3

bisbas5_w2 | J1*t1 + J2*t2 + J3*t3

bisbas10_w2 | K1*t1 + K2*t2 + K3*t3

bisbas15_w2 | L1*t1 + L2*t2 + L3*t3

bisbas20_w2 | M1*t1 + M2*t2 + M3*t3

bisbas5_w3 | J1*t1 + J2*t2 + J3*t3

bisbas10_w3 | K1*t1 + K2*t2 + K3*t3

bisbas15_w3 | L1*t1 + L2*t2 + L3*t3

bisbas20_w3 | M1*t1 + M2*t2 + M3*t3

bisbas5_w4 | J1*t1 + J2*t2 + J3*t3

bisbas10_w4 | K1*t1 + K2*t2 + K3*t3

bisbas15_w4 | L1*t1 + L2*t2 + L3*t3

bisbas20_w4 | M1*t1 + M2*t2 + M3*t3

# BAS_r

bisbas4_w1 | N1*t1 + N2*t2 + N3*t3

bisbas7_w1 | O1*t1 + O2*t2 + O3*t3

bisbas14_w1 | P1*t1 + P2*t2 + P3*t3

bisbas18_w1 | Q1*t1 + Q2*t2 + Q3*t3

bisbas23_w1 | R1*t1 + R2*t2 + R3*t3

bisbas4_w2 | N1*t1 + N2*t2 + N3*t3

bisbas7_w2 | O1*t1 + O2*t2 + O3*t3

bisbas14_w2 | P1*t1 + P2*t2 + P3*t3

bisbas18_w2 | Q1*t1 + Q2*t2 + Q3*t3

bisbas23_w2 | R1*t1 + R2*t2 + R3*t3

bisbas4_w3 | N1*t1 + N2*t2 + N3*t3

bisbas7_w3 | O1*t1 + O2*t2 + O3*t3

bisbas14_w3 | P1*t1 + P2*t2 + P3*t3

bisbas18_w3 | Q1*t1 + Q2*t2 + Q3*t3

bisbas23_w3 | R1*t1 + R2*t2 + R3*t3

bisbas4_w4 | N1*t1 + N2*t2 + N3*t3

bisbas7_w4 | O1*t1 + O2*t2 + O3*t3

bisbas14_w4 | P1*t1 + P2*t2 + P3*t3

bisbas18_w4 | Q1*t1 + Q2*t2 + Q3*t3

bisbas23_w4 | R1*t1 + R2*t2 + R3*t3

# FFFS

bisbas22_w1R | S1*t1 + S2*t2 + S3*t3

bisbas2_w1R | T1*t1 + T2*t2 + T3*t3

bisbas22_w2R | S1*t1 + S2*t2 + S3*t3

bisbas2_w2R | T1*t1 + T2*t2 + T3*t3

bisbas22_w3R | S1*t1 + S2*t2 + S3*t3

bisbas2_w3R | T1*t1 + T2*t2 + T3*t3

bisbas22_w4R | S1*t1 + S2*t2 + S3*t3

bisbas2_w4R | T1*t1 + T2*t2 + T3*t3

# All variable variances are set to one at wave 1, free at

other waves

# BIS

bisbas8_w1 ~~ 1*bisbas8_w1

bisbas8_w2 ~~ NA*bisbas8_w2

bisbas8_w3 ~~ NA*bisbas8_w3

bisbas8_w4 ~~ NA*bisbas8_w4

bisbas13_w1 ~~ 1*bisbas13_w1

bisbas13_w2 ~~ NA*bisbas13_w2

bisbas13_w3 ~~ NA*bisbas13_w3

bisbas13_w4 ~~ NA*bisbas13_w4

bisbas16_w1 ~~ 1*bisbas16_w1

bisbas16_w2 ~~ NA*bisbas16_w2

bisbas16_w3 ~~ NA*bisbas16_w3

bisbas16_w4 ~~ NA*bisbas16_w4

bisbas19_w1 ~~ 1*bisbas19_w1

bisbas19_w2 ~~ NA*bisbas19_w2

bisbas19_w3 ~~ NA*bisbas19_w3

bisbas19_w4 ~~ NA*bisbas19_w4

bisbas24_w1 ~~ 1*bisbas24_w1

bisbas24_w2 ~~ NA*bisbas24_w2

bisbas24_w3 ~~ NA*bisbas24_w3

bisbas24_w4 ~~ NA*bisbas24_w4

# BAS-d

bisbas3_w1 ~~ 1*bisbas3_w1

bisbas3_w2 ~~ NA*bisbas3_w2

bisbas3_w3 ~~ NA*bisbas3_w3

bisbas3_w4 ~~ NA*bisbas3_w4

bisbas9_w1 ~~ 1*bisbas9_w1

bisbas9_w2 ~~ NA*bisbas9_w2

bisbas9_w3 ~~ NA*bisbas9_w3

bisbas9_w4 ~~ NA*bisbas9_w4

bisbas12_w1 ~~ 1*bisbas12_w1

bisbas12_w2 ~~ NA*bisbas12_w2

bisbas12_w3 ~~ NA*bisbas12_w3

bisbas12_w4 ~~ NA*bisbas12_w4

bisbas21_w1 ~~ 1*bisbas21_w1

bisbas21_w2 ~~ NA*bisbas21_w2

bisbas21_w3 ~~ NA*bisbas21_w3

bisbas21_w4 ~~ NA*bisbas21_w4

# BAS_f

bisbas5_w1 ~~ 1*bisbas5_w1

bisbas5_w2 ~~ NA*bisbas5_w2

bisbas5_w3 ~~ NA*bisbas5_w3

bisbas5_w4 ~~ NA*bisbas5_w4

bisbas10_w1 ~~ 1*bisbas10_w1

bisbas10_w2 ~~ NA*bisbas10_w2

bisbas10_w3 ~~ NA*bisbas10_w3

bisbas10_w4 ~~ NA*bisbas10_w4

bisbas15_w1 ~~ 1*bisbas15_w1

bisbas15_w2 ~~ NA*bisbas15_w2

bisbas15_w3 ~~ NA*bisbas15_w3

bisbas15_w4 ~~ NA*bisbas15_w4

bisbas20_w1 ~~ 1*bisbas20_w1

bisbas20_w2 ~~ NA*bisbas20_w2

bisbas20_w3 ~~ NA*bisbas20_w3

bisbas20_w4 ~~ NA*bisbas20_w4

# BAS_r

bisbas4_w1 ~~ 1*bisbas4_w1

bisbas4_w2 ~~ NA*bisbas4_w2

bisbas4_w3 ~~ NA*bisbas4_w3

bisbas4_w4 ~~ NA*bisbas4_w4

bisbas7_w1 ~~ 1*bisbas7_w1

bisbas7_w2 ~~ NA*bisbas7_w2

bisbas7_w3 ~~ NA*bisbas7_w3

bisbas7_w4 ~~ NA*bisbas7_w4

bisbas14_w1 ~~ 1*bisbas14_w1

bisbas14_w2 ~~ NA*bisbas14_w2

bisbas14_w3 ~~ NA*bisbas14_w3

bisbas14_w4 ~~ NA*bisbas14_w4

bisbas18_w1 ~~ 1*bisbas18_w1

bisbas18_w2 ~~ NA*bisbas18_w2

bisbas18_w3 ~~ NA*bisbas18_w3

bisbas18_w4 ~~ NA*bisbas18_w4

bisbas23_w1 ~~ 1*bisbas23_w1

bisbas23_w2 ~~ NA*bisbas23_w2

bisbas23_w3 ~~ NA*bisbas23_w3

bisbas23_w4 ~~ NA*bisbas23_w4

# FFFS

bisbas22_w1R ~~ 1*bisbas22_w1R

bisbas22_w2R ~~ NA*bisbas22_w2R

bisbas22_w3R ~~ NA*bisbas22_w3R

bisbas22_w4R ~~ NA*bisbas22_w4R

bisbas2_w1R ~~ 1*bisbas2_w1R

bisbas2_w2R ~~ NA*bisbas2_w2R

bisbas2_w3R ~~ NA*bisbas2_w3R

bisbas2_w4R ~~ NA*bisbas2_w4R

# Unique factor covariances across waves

# BIS

bisbas8_w1 ~~ bisbas8_w2 + bisbas8_w3 + bisbas8_w4

bisbas8_w2 ~~ bisbas8_w3 + bisbas8_w4

bisbas8_w3 ~~ bisbas8_w4

bisbas13_w1 ~~ bisbas13_w2 + bisbas13_w3 + bisbas13_w4

bisbas13_w2 ~~ bisbas13_w3 + bisbas13_w4

bisbas13_w3 ~~ bisbas13_w4

bisbas16_w1 ~~ bisbas16_w2 + bisbas16_w3 + bisbas16_w4

bisbas16_w2 ~~ bisbas16_w3 + bisbas16_w4

bisbas16_w3 ~~ bisbas16_w4

bisbas19_w1 ~~ bisbas19_w2 + bisbas19_w3 + bisbas19_w4

bisbas19_w2 ~~ bisbas19_w3 + bisbas19_w4

bisbas19_w3 ~~ bisbas19_w4

bisbas24_w1 ~~ bisbas24_w2 + bisbas24_w3 + bisbas24_w4

bisbas24_w2 ~~ bisbas24_w3 + bisbas24_w4

bisbas24_w3 ~~ bisbas24_w4

# BAS_d

bisbas3_w1 ~~ bisbas3_w2 + bisbas3_w3 + bisbas3_w4

bisbas3_w2 ~~ bisbas3_w3 + bisbas3_w4

bisbas3_w3 ~~ bisbas3_w4

bisbas9_w1 ~~ bisbas9_w2 + bisbas9_w3 + bisbas9_w4

bisbas9_w2 ~~ bisbas9_w3 + bisbas9_w4

bisbas9_w3 ~~ bisbas9_w4

bisbas12_w1 ~~ bisbas12_w2 + bisbas12_w3 + bisbas12_w4

bisbas12_w2 ~~ bisbas12_w3 + bisbas12_w4

bisbas12_w3 ~~ bisbas12_w4

bisbas21_w1 ~~ bisbas21_w2 + bisbas21_w3 + bisbas21_w4

bisbas21_w2 ~~ bisbas21_w3 + bisbas21_w4

bisbas21_w3 ~~ bisbas21_w4

# BAS_f

bisbas5_w1 ~~ bisbas5_w2 + bisbas5_w3 + bisbas5_w4

bisbas5_w2 ~~ bisbas5_w3 + bisbas5_w4

bisbas5_w3 ~~ bisbas5_w4

bisbas10_w1 ~~ bisbas10_w2 + bisbas10_w3 + bisbas10_w4

bisbas10_w2 ~~ bisbas10_w3 + bisbas10_w4

bisbas10_w3 ~~ bisbas10_w4

bisbas15_w1 ~~ bisbas15_w2 + bisbas15_w3 + bisbas15_w4

bisbas15_w2 ~~ bisbas15_w3 + bisbas15_w4

bisbas15_w3 ~~ bisbas15_w4

bisbas20_w1 ~~ bisbas20_w2 + bisbas20_w3 + bisbas20_w4

bisbas20_w2 ~~ bisbas20_w3 + bisbas20_w4

bisbas20_w3 ~~ bisbas20_w4

# BAS_r

bisbas4_w1 ~~ bisbas4_w2 + bisbas4_w3 + bisbas4_w4

bisbas4_w2 ~~ bisbas4_w3 + bisbas4_w4

bisbas4_w3 ~~ bisbas4_w4

bisbas7_w1 ~~ bisbas7_w2 + bisbas7_w3 + bisbas7_w4

bisbas7_w2 ~~ bisbas7_w3 + bisbas7_w4

bisbas7_w3 ~~ bisbas7_w4

bisbas14_w1 ~~ bisbas14_w2 + bisbas14_w3 + bisbas14_w4

bisbas14_w2 ~~ bisbas14_w3 + bisbas14_w4

bisbas14_w3 ~~ bisbas14_w4

bisbas18_w1 ~~ bisbas18_w2 + bisbas18_w3 + bisbas18_w4

bisbas18_w2 ~~ bisbas18_w3 + bisbas18_w4

bisbas18_w3 ~~ bisbas18_w4

bisbas23_w1 ~~ bisbas23_w2 + bisbas23_w3 + bisbas23_w4

bisbas23_w2 ~~ bisbas23_w3 + bisbas23_w4

bisbas23_w3 ~~ bisbas23_w4

# FFFS

bisbas22_w1R ~~ bisbas22_w2R + bisbas22_w3R + bisbas22_w4R

bisbas22_w2R ~~ bisbas22_w3R + bisbas22_w4R

bisbas22_w3R ~~ bisbas22_w4R

bisbas2_w1R ~~ bisbas2_w2R + bisbas2_w3R + bisbas2_w4R

bisbas2_w2R ~~ bisbas2_w3R + bisbas2_w4R

bisbas2_w3R ~~ bisbas2_w4R'

#

# Fit model:

#

fit_Emi.bb_model2 <- cfa(Emi.bb_model2, data = PATH, ordered = c("bisbas8_w1", "bisbas8_w2", "bisbas8_w3", "bisbas8_w4", "bisbas13_w1", "bisbas13_w2", "bisbas13_w3", "bisbas13_w4", "bisbas16_w1", "bisbas16_w2", "bisbas16_w3", "bisbas16_w4", "bisbas19_w1", "bisbas19_w2", "bisbas19_w3", "bisbas19_w4", "bisbas24_w1", "bisbas24_w2", "bisbas24_w3", "bisbas24_w4", "bisbas3_w1", "bisbas3_w2", "bisbas3_w3", "bisbas3_w4", "bisbas9_w1", "bisbas9_w2", "bisbas9_w3", "bisbas9_w4", "bisbas12_w1", "bisbas12_w2", "bisbas12_w3", "bisbas12_w4", "bisbas21_w1", "bisbas21_w2", "bisbas21_w3", "bisbas21_w4", "bisbas5_w1", "bisbas5_w2", "bisbas5_w3", "bisbas5_w4", "bisbas10_w1", "bisbas10_w2", "bisbas10_w3", "bisbas10_w4", "bisbas15_w1", "bisbas15_w2", "bisbas15_w3", "bisbas15_w4", "bisbas20_w1", "bisbas20_w2", "bisbas20_w3", "bisbas20_w4", "bisbas4_w1", "bisbas4_w2", "bisbas4_w3", "bisbas4_w4", "bisbas7_w1", "bisbas7_w2", "bisbas7_w3", "bisbas7_w4", "bisbas14_w1", "bisbas14_w2", "bisbas14_w3", "bisbas14_w4", "bisbas18_w1", "bisbas18_w2", "bisbas18_w3", "bisbas18_w4", "bisbas23_w1", "bisbas23_w2", "bisbas23_w3", "bisbas23_w4", "bisbas22_w1R", "bisbas22_w2R", "bisbas22_w3R", "bisbas22_w4R", "bisbas2_w1R", "bisbas2_w2R", "bisbas2_w3R", "bisbas2_w4R"), parameterization = "theta", missing = "pairwise", mimic = "Mplus")

#

# Show fit measures:

#

summary(fit_Emi.bb_model2, fit.measures = TRUE)

**Loading Invariance**

# Model definition:

#

Emi.bb_model3 <- '# Latent factors, fixing loadings to be equal at all

waves

BIS1 =~ bisbas8_w1 + a*bisbas13_w1 + b*bisbas16_w1

+ c*bisbas19_w1+ d*bisbas24_w1

BIS2 =~ bisbas8_w2 + a*bisbas13_w2 + b*bisbas16_w2

+ c*bisbas19_w2+ d*bisbas24_w2

BIS3 =~ bisbas8_w3 + a*bisbas13_w3 + b*bisbas16_w3

+ c*bisbas19_w3+ d*bisbas24_w3

BIS4 =~ bisbas8_w4 + a*bisbas13_w4 + b*bisbas16_w4

+ c*bisbas19_w4+ d*bisbas24_w4

BAS_d1 =~ bisbas3_w1 + e*bisbas9_w1 + f*bisbas12_w1

+ g*bisbas21_w1

BAS_d2 =~ bisbas3_w2 + e*bisbas9_w2 + f*bisbas12_w2

+ g*bisbas21_w2

BAS_d3 =~ bisbas3_w3 + e*bisbas9_w3 + f*bisbas12_w3

+ g*bisbas21_w3

BAS_d4 =~ bisbas3_w4 + e*bisbas9_w4 + f*bisbas12_w4

+ g*bisbas21_w4

BAS_f1 =~ bisbas5_w1 + h*bisbas10_w1 + i*bisbas15_w1

+ j*bisbas20_w1

BAS_f2 =~ bisbas5_w2 + h*bisbas10_w2 + i*bisbas15_w2

+ j*bisbas20_w2

BAS_f3 =~ bisbas5_w3 + h*bisbas10_w3 + i*bisbas15_w3

+ j*bisbas20_w3

BAS_f4 =~ bisbas5_w4 + h*bisbas10_w4 + i*bisbas15_w4

+ j*bisbas20_w4

BAS_r1 =~ bisbas4_w1 + k*bisbas7_w1 + l*bisbas14_w1

+ m*bisbas18_w1 + n*bisbas23_w1

BAS_r2 =~ bisbas4_w2 + k*bisbas7_w2 + l*bisbas14_w2

+ m*bisbas18_w2 + n*bisbas23_w2

BAS_r3 =~ bisbas4_w3 + k*bisbas7_w3 + l*bisbas14_w3

+ m*bisbas18_w3 + n*bisbas23_w3

BAS_r4 =~ bisbas4_w4 + k*bisbas7_w4 + l*bisbas14_w4

+ m*bisbas18_w4 + n*bisbas23_w4

FFFS1 =~ bisbas22_w1R + o*bisbas2_w1R

FFFS2 =~ bisbas22_w2R + o*bisbas2_w2R

FFFS3 =~ bisbas22_w3R + o*bisbas2_w3R

FFFS4 =~ bisbas22_w4R + o*bisbas2_w4R

# Latent factor covariances across waves

BIS1 ~~ BIS2 + BIS3 + BIS4

BIS2 ~~ BIS3 + BIS4

BIS3 ~~ BIS4

BAS_d1 ~~ BAS_d2 + BAS_d3 + BAS_d4

BAS_d2 ~~ BAS_d3 + BAS_d4

BAS_d3 ~~ BAS_d4

BAS_f1 ~~ BAS_f2 + BAS_f3 + BAS_f4

BAS_f2 ~~ BAS_f3 + BAS_f4

BAS_f3 ~~ BAS_f4

BAS_r1 ~~ BAS_r2 + BAS_r3 + BAS_r4

BAS_r2 ~~ BAS_r3 + BAS_r4

BAS_r3 ~~ BAS_r4

FFFS1 ~~ FFFS2 + FFFS3 + FFFS4

FFFS2 ~~ FFFS3 + FFFS4

FFFS3 ~~ FFFS4

# Latent factor covariances within waves

# Wave 1

BIS1 ~~ BAS_d1 + BAS_f1 + BAS_r1 + FFFS1

BAS_d1 ~~ BAS_f1 + BAS_r1 + FFFS1

BAS_f1 ~~ BAS_r1 + FFFS1

BAS_r1 ~~ FFFS1

# Wave 2

BIS2 ~~ BAS_d2 + BAS_f2 + BAS_r2 + FFFS2

BAS_d2 ~~ BAS_f2 + BAS_r2 + FFFS2

BAS_f2 ~~ BAS_r2 + FFFS2

BAS_r2 ~~ FFFS2

# Wave 3

BIS3 ~~ BAS_d3 + BAS_f3 + BAS_r3 + FFFS3

BAS_d3 ~~ BAS_f3 + BAS_r3 + FFFS3

BAS_f3 ~~ BAS_r3 + FFFS3

BAS_r3 ~~ FFFS3

# Wave 4

BIS4 ~~ BAS_d4 + BAS_f4 + BAS_r4 + FFFS4

BAS_d4 ~~ BAS_f4 + BAS_r4 + FFFS4

BAS_f4 ~~ BAS_r4 + FFFS4

BAS_r4 ~~ FFFS4

# Latent factor means

# Fix mean to zero only at wave 1, free at all other waves

BIS1 ~ 0*1

BIS2 ~ NA*1

BIS3 ~ NA*1

BIS4 ~ NA*1

BAS_d1 ~ 0*1

BAS_d2 ~ NA*1

BAS_d3 ~ NA*1

BAS_d4 ~ NA*1

BAS_f1 ~ 0*1

BAS_f2 ~ NA*1

BAS_f3 ~ NA*1

BAS_f4 ~ NA*1

BAS_r1 ~ 0*1

BAS_r2 ~ NA*1

BAS_r3 ~ NA*1

BAS_r4 ~ NA*1

FFFS1 ~ 0*1

FFFS2 ~ NA*1

FFFS3 ~ NA*1

FFFS4 ~ NA*1

# All intercepts are set to zero as part of model definition

by lavaan

# Fix thresholds to be identical between waves

# BIS

bisbas8_w1 | A1*t1 + A2*t2 + A3*t3

bisbas13_w1 | B1*t1 + B2*t2 + B3*t3

bisbas16_w1 | C1*t1 + C2*t2 + C3*t3

bisbas19_w1 | D1*t1 + D2*t2 + D3*t3

bisbas24_w1 | E1*t1 + E2*t2 + E3*t3

bisbas8_w2 | A1*t1 + A2*t2 + A3*t3

bisbas13_w2 | B1*t1 + B2*t2 + B3*t3

bisbas16_w2 | C1*t1 + C2*t2 + C3*t3

bisbas19_w2 | D1*t1 + D2*t2 + D3*t3

bisbas24_w2 | E1*t1 + E2*t2 + E3*t3

bisbas8_w3 | A1*t1 + A2*t2 + A3*t3

bisbas13_w3 | B1*t1 + B2*t2 + B3*t3

bisbas16_w3 | C1*t1 + C2*t2 + C3*t3

bisbas19_w3 | D1*t1 + D2*t2 + D3*t3

bisbas24_w3 | E1*t1 + E2*t2 + E3*t3

bisbas8_w4 | A1*t1 + A2*t2 + A3*t3

bisbas13_w4 | B1*t1 + B2*t2 + B3*t3

bisbas16_w4 | C1*t1 + C2*t2 + C3*t3

bisbas19_w4 | D1*t1 + D2*t2 + D3*t3

bisbas24_w4 | E1*t1 + E2*t2 + E3*t3

# BAS_d

bisbas3_w1 | F1*t1 + F2*t2 + F3*t3

bisbas9_w1 | G1*t1 + G2*t2 + G3*t3

bisbas12_w1 | H1*t1 + H2*t2 + H3*t3

bisbas21_w1 | I1*t1 + I2*t2 + I3*t3

bisbas3_w2 | F1*t1 + F2*t2 + F3*t3

bisbas9_w2 | G1*t1 + G2*t2 + G3*t3

bisbas12_w2 | H1*t1 + H2*t2 + H3*t3

bisbas21_w2 | I1*t1 + I2*t2 + I3*t3

bisbas3_w3 | F1*t1 + F2*t2 + F3*t3

bisbas9_w3 | G1*t1 + G2*t2 + G3*t3

bisbas12_w3 | H1*t1 + H2*t2 + H3*t3

bisbas21_w3 | I1*t1 + I2*t2 + I3*t3

bisbas3_w4 | F1*t1 + F2*t2 + F3*t3

bisbas9_w4 | G1*t1 + G2*t2 + G3*t3

bisbas12_w4 | H1*t1 + H2*t2 + H3*t3

bisbas21_w4 | I1*t1 + I2*t2 + I3*t3

# BAS_f

bisbas5_w1 | J1*t1 + J2*t2 + J3*t3

bisbas10_w1 | K1*t1 + K2*t2 + K3*t3

bisbas15_w1 | L1*t1 + L2*t2 + L3*t3

bisbas20_w1 | M1*t1 + M2*t2 + M3*t3

bisbas5_w2 | J1*t1 + J2*t2 + J3*t3

bisbas10_w2 | K1*t1 + K2*t2 + K3*t3

bisbas15_w2 | L1*t1 + L2*t2 + L3*t3

bisbas20_w2 | M1*t1 + M2*t2 + M3*t3

bisbas5_w3 | J1*t1 + J2*t2 + J3*t3

bisbas10_w3 | K1*t1 + K2*t2 + K3*t3

bisbas15_w3 | L1*t1 + L2*t2 + L3*t3

bisbas20_w3 | M1*t1 + M2*t2 + M3*t3

bisbas5_w4 | J1*t1 + J2*t2 + J3*t3

bisbas10_w4 | K1*t1 + K2*t2 + K3*t3

bisbas15_w4 | L1*t1 + L2*t2 + L3*t3

bisbas20_w4 | M1*t1 + M2*t2 + M3*t3

# BAS_r

bisbas4_w1 | N1*t1 + N2*t2 + N3*t3

bisbas7_w1 | O1*t1 + O2*t2 + O3*t3

bisbas14_w1 | P1*t1 + P2*t2 + P3*t3

bisbas18_w1 | Q1*t1 + Q2*t2 + Q3*t3

bisbas23_w1 | R1*t1 + R2*t2 + R3*t3

bisbas4_w2 | N1*t1 + N2*t2 + N3*t3

bisbas7_w2 | O1*t1 + O2*t2 + O3*t3

bisbas14_w2 | P1*t1 + P2*t2 + P3*t3

bisbas18_w2 | Q1*t1 + Q2*t2 + Q3*t3

bisbas23_w2 | R1*t1 + R2*t2 + R3*t3

bisbas4_w3 | N1*t1 + N2*t2 + N3*t3

bisbas7_w3 | O1*t1 + O2*t2 + O3*t3

bisbas14_w3 | P1*t1 + P2*t2 + P3*t3

bisbas18_w3 | Q1*t1 + Q2*t2 + Q3*t3

bisbas23_w3 | R1*t1 + R2*t2 + R3*t3

bisbas4_w4 | N1*t1 + N2*t2 + N3*t3

bisbas7_w4 | O1*t1 + O2*t2 + O3*t3

bisbas14_w4 | P1*t1 + P2*t2 + P3*t3

bisbas18_w4 | Q1*t1 + Q2*t2 + Q3*t3

bisbas23_w4 | R1*t1 + R2*t2 + R3*t3

# FFFS

bisbas22_w1R | S1*t1 + S2*t2 + S3*t3

bisbas2_w1R | T1*t1 + T2*t2 + T3*t3

bisbas22_w2R | S1*t1 + S2*t2 + S3*t3

bisbas2_w2R | T1*t1 + T2*t2 + T3*t3

bisbas22_w3R | S1*t1 + S2*t2 + S3*t3

bisbas2_w3R | T1*t1 + T2*t2 + T3*t3

bisbas22_w4R | S1*t1 + S2*t2 + S3*t3

bisbas2_w4R | T1*t1 + T2*t2 + T3*t3

# All variable variances are set to one at wave 1, free at

other waves

# BIS

bisbas8_w1 ~~ 1*bisbas8_w1

bisbas8_w2 ~~ NA*bisbas8_w2

bisbas8_w3 ~~ NA*bisbas8_w3

bisbas8_w4 ~~ NA*bisbas8_w4

bisbas13_w1 ~~ 1*bisbas13_w1

bisbas13_w2 ~~ NA*bisbas13_w2

bisbas13_w3 ~~ NA*bisbas13_w3

bisbas13_w4 ~~ NA*bisbas13_w4

bisbas16_w1 ~~ 1*bisbas16_w1

bisbas16_w2 ~~ NA*bisbas16_w2

bisbas16_w3 ~~ NA*bisbas16_w3

bisbas16_w4 ~~ NA*bisbas16_w4

bisbas19_w1 ~~ 1*bisbas19_w1

bisbas19_w2 ~~ NA*bisbas19_w2

bisbas19_w3 ~~ NA*bisbas19_w3

bisbas19_w4 ~~ NA*bisbas19_w4

bisbas24_w1 ~~ 1*bisbas24_w1

bisbas24_w2 ~~ NA*bisbas24_w2

bisbas24_w3 ~~ NA*bisbas24_w3

bisbas24_w4 ~~ NA*bisbas24_w4

# BAS-d

bisbas3_w1 ~~ 1*bisbas3_w1

bisbas3_w2 ~~ NA*bisbas3_w2

bisbas3_w3 ~~ NA*bisbas3_w3

bisbas3_w4 ~~ NA*bisbas3_w4

bisbas9_w1 ~~ 1*bisbas9_w1

bisbas9_w2 ~~ NA*bisbas9_w2

bisbas9_w3 ~~ NA*bisbas9_w3

bisbas9_w4 ~~ NA*bisbas9_w4

bisbas12_w1 ~~ 1*bisbas12_w1

bisbas12_w2 ~~ NA*bisbas12_w2

bisbas12_w3 ~~ NA*bisbas12_w3

bisbas12_w4 ~~ NA*bisbas12_w4

bisbas21_w1 ~~ 1*bisbas21_w1

bisbas21_w2 ~~ NA*bisbas21_w2

bisbas21_w3 ~~ NA*bisbas21_w3

bisbas21_w4 ~~ NA*bisbas21_w4

# BAS_f

bisbas5_w1 ~~ 1*bisbas5_w1

bisbas5_w2 ~~ NA*bisbas5_w2

bisbas5_w3 ~~ NA*bisbas5_w3

bisbas5_w4 ~~ NA*bisbas5_w4

bisbas10_w1 ~~ 1*bisbas10_w1

bisbas10_w2 ~~ NA*bisbas10_w2

bisbas10_w3 ~~ NA*bisbas10_w3

bisbas10_w4 ~~ NA*bisbas10_w4

bisbas15_w1 ~~ 1*bisbas15_w1

bisbas15_w2 ~~ NA*bisbas15_w2

bisbas15_w3 ~~ NA*bisbas15_w3

bisbas15_w4 ~~ NA*bisbas15_w4

bisbas20_w1 ~~ 1*bisbas20_w1

bisbas20_w2 ~~ NA*bisbas20_w2

bisbas20_w3 ~~ NA*bisbas20_w3

bisbas20_w4 ~~ NA*bisbas20_w4

# BAS_r

bisbas4_w1 ~~ 1*bisbas4_w1

bisbas4_w2 ~~ NA*bisbas4_w2

bisbas4_w3 ~~ NA*bisbas4_w3

bisbas4_w4 ~~ NA*bisbas4_w4

bisbas7_w1 ~~ 1*bisbas7_w1

bisbas7_w2 ~~ NA*bisbas7_w2

bisbas7_w3 ~~ NA*bisbas7_w3

bisbas7_w4 ~~ NA*bisbas7_w4

bisbas14_w1 ~~ 1*bisbas14_w1

bisbas14_w2 ~~ NA*bisbas14_w2

bisbas14_w3 ~~ NA*bisbas14_w3

bisbas14_w4 ~~ NA*bisbas14_w4

bisbas18_w1 ~~ 1*bisbas18_w1

bisbas18_w2 ~~ NA*bisbas18_w2

bisbas18_w3 ~~ NA*bisbas18_w3

bisbas18_w4 ~~ NA*bisbas18_w4

bisbas23_w1 ~~ 1*bisbas23_w1

bisbas23_w2 ~~ NA*bisbas23_w2

bisbas23_w3 ~~ NA*bisbas23_w3

bisbas23_w4 ~~ NA*bisbas23_w4

# FFFS

bisbas22_w1R ~~ 1*bisbas22_w1R

bisbas22_w2R ~~ NA*bisbas22_w2R

bisbas22_w3R ~~ NA*bisbas22_w3R

bisbas22_w4R ~~ NA*bisbas22_w4R

bisbas2_w1R ~~ 1*bisbas2_w1R

bisbas2_w2R ~~ NA*bisbas2_w2R

bisbas2_w3R ~~ NA*bisbas2_w3R

bisbas2_w4R ~~ NA*bisbas2_w4R

# Unique factor covariances across waves

# BIS

bisbas8_w1 ~~ bisbas8_w2 + bisbas8_w3 + bisbas8_w4

bisbas8_w2 ~~ bisbas8_w3 + bisbas8_w4

bisbas8_w3 ~~ bisbas8_w4

bisbas13_w1 ~~ bisbas13_w2 + bisbas13_w3 + bisbas13_w4

bisbas13_w2 ~~ bisbas13_w3 + bisbas13_w4

bisbas13_w3 ~~ bisbas13_w4

bisbas16_w1 ~~ bisbas16_w2 + bisbas16_w3 + bisbas16_w4

bisbas16_w2 ~~ bisbas16_w3 + bisbas16_w4

bisbas16_w3 ~~ bisbas16_w4

bisbas19_w1 ~~ bisbas19_w2 + bisbas19_w3 + bisbas19_w4

bisbas19_w2 ~~ bisbas19_w3 + bisbas19_w4

bisbas19_w3 ~~ bisbas19_w4

bisbas24_w1 ~~ bisbas24_w2 + bisbas24_w3 + bisbas24_w4

bisbas24_w2 ~~ bisbas24_w3 + bisbas24_w4

bisbas24_w3 ~~ bisbas24_w4

# BAS_d

bisbas3_w1 ~~ bisbas3_w2 + bisbas3_w3 + bisbas3_w4

bisbas3_w2 ~~ bisbas3_w3 + bisbas3_w4

bisbas3_w3 ~~ bisbas3_w4

bisbas9_w1 ~~ bisbas9_w2 + bisbas9_w3 + bisbas9_w4

bisbas9_w2 ~~ bisbas9_w3 + bisbas9_w4

bisbas9_w3 ~~ bisbas9_w4

bisbas12_w1 ~~ bisbas12_w2 + bisbas12_w3 + bisbas12_w4

bisbas12_w2 ~~ bisbas12_w3 + bisbas12_w4

bisbas12_w3 ~~ bisbas12_w4

bisbas21_w1 ~~ bisbas21_w2 + bisbas21_w3 + bisbas21_w4

bisbas21_w2 ~~ bisbas21_w3 + bisbas21_w4

bisbas21_w3 ~~ bisbas21_w4

# BAS_f

bisbas5_w1 ~~ bisbas5_w2 + bisbas5_w3 + bisbas5_w4

bisbas5_w2 ~~ bisbas5_w3 + bisbas5_w4

bisbas5_w3 ~~ bisbas5_w4

bisbas10_w1 ~~ bisbas10_w2 + bisbas10_w3 + bisbas10_w4

bisbas10_w2 ~~ bisbas10_w3 + bisbas10_w4

bisbas10_w3 ~~ bisbas10_w4

bisbas15_w1 ~~ bisbas15_w2 + bisbas15_w3 + bisbas15_w4

bisbas15_w2 ~~ bisbas15_w3 + bisbas15_w4

bisbas15_w3 ~~ bisbas15_w4

bisbas20_w1 ~~ bisbas20_w2 + bisbas20_w3 + bisbas20_w4

bisbas20_w2 ~~ bisbas20_w3 + bisbas20_w4

bisbas20_w3 ~~ bisbas20_w4

# BAS_r

bisbas4_w1 ~~ bisbas4_w2 + bisbas4_w3 + bisbas4_w4

bisbas4_w2 ~~ bisbas4_w3 + bisbas4_w4

bisbas4_w3 ~~ bisbas4_w4

bisbas7_w1 ~~ bisbas7_w2 + bisbas7_w3 + bisbas7_w4

bisbas7_w2 ~~ bisbas7_w3 + bisbas7_w4

bisbas7_w3 ~~ bisbas7_w4

bisbas14_w1 ~~ bisbas14_w2 + bisbas14_w3 + bisbas14_w4

bisbas14_w2 ~~ bisbas14_w3 + bisbas14_w4

bisbas14_w3 ~~ bisbas14_w4

bisbas18_w1 ~~ bisbas18_w2 + bisbas18_w3 + bisbas18_w4

bisbas18_w2 ~~ bisbas18_w3 + bisbas18_w4

bisbas18_w3 ~~ bisbas18_w4

bisbas23_w1 ~~ bisbas23_w2 + bisbas23_w3 + bisbas23_w4

bisbas23_w2 ~~ bisbas23_w3 + bisbas23_w4

bisbas23_w3 ~~ bisbas23_w4

# FFFS

bisbas22_w1R ~~ bisbas22_w2R + bisbas22_w3R + bisbas22_w4R

bisbas22_w2R ~~ bisbas22_w3R + bisbas22_w4R

bisbas22_w3R ~~ bisbas22_w4R

bisbas2_w1R ~~ bisbas2_w2R + bisbas2_w3R + bisbas2_w4R

bisbas2_w2R ~~ bisbas2_w3R + bisbas2_w4R

bisbas2_w3R ~~ bisbas2_w4R'

#

# Fit model:

#

fit_Emi.bb_model3 <- cfa(Emi.bb_model3, data = PATH, ordered = c("bisbas8_w1", "bisbas8_w2", "bisbas8_w3", "bisbas8_w4", "bisbas13_w1", "bisbas13_w2", "bisbas13_w3", "bisbas13_w4", "bisbas16_w1", "bisbas16_w2", "bisbas16_w3", "bisbas16_w4", "bisbas19_w1", "bisbas19_w2", "bisbas19_w3", "bisbas19_w4", "bisbas24_w1", "bisbas24_w2", "bisbas24_w3", "bisbas24_w4", "bisbas3_w1", "bisbas3_w2", "bisbas3_w3", "bisbas3_w4", "bisbas9_w1", "bisbas9_w2", "bisbas9_w3", "bisbas9_w4", "bisbas12_w1", "bisbas12_w2", "bisbas12_w3", "bisbas12_w4", "bisbas21_w1", "bisbas21_w2", "bisbas21_w3", "bisbas21_w4", "bisbas5_w1", "bisbas5_w2", "bisbas5_w3", "bisbas5_w4", "bisbas10_w1", "bisbas10_w2", "bisbas10_w3", "bisbas10_w4", "bisbas15_w1", "bisbas15_w2", "bisbas15_w3", "bisbas15_w4", "bisbas20_w1", "bisbas20_w2", "bisbas20_w3", "bisbas20_w4", "bisbas4_w1", "bisbas4_w2", "bisbas4_w3", "bisbas4_w4", "bisbas7_w1", "bisbas7_w2", "bisbas7_w3", "bisbas7_w4", "bisbas14_w1", "bisbas14_w2", "bisbas14_w3", "bisbas14_w4", "bisbas18_w1", "bisbas18_w2", "bisbas18_w3", "bisbas18_w4", "bisbas23_w1", "bisbas23_w2", "bisbas23_w3", "bisbas23_w4", "bisbas22_w1R", "bisbas22_w2R", "bisbas22_w3R", "bisbas22_w4R", "bisbas2_w1R", "bisbas2_w2R", "bisbas2_w3R", "bisbas2_w4R"), parameterization = "theta", missing = "pairwise", mimic = "Mplus")

#

# Show fit measures:

#

summary(fit_Emi.bb_model3, fit.measures = TRUE)

**Alternative approach to measurement invariance**

The measurement invariance approach proposed by Muthén and Muthén (Muthén and Muthén, 1998-2011), contrasting configural and loading nested models, was also implemented. As expected significant *χ^2^* tests were found, but there was low variation in fit indices: age (*Δχ^2^* = 521.97, *Δdf* = 100, *p* < .001, *ΔRMSEA* = -.002, *ΔCFI* = .008), sex (*Δχ^2^* = 240.70, *Δdf* = 50, *p* < .001, *ΔRMSEA* = .001, *ΔCFI* = .003), and longitudinal (*Δχ^2^* = 1319.80, *Δdf* = 150, *p* < .001, *ΔRMSEA* = .000, *ΔCFI* = .001). Therefore confirming measurement invariance under these conditions.

# Supplementary Tables

**Supplement Table 1. Descriptive statistics of PATH wave 1 subsamples.** BIS: Behavioural inhibition, BAS: Behavioural approach/activation, BAS-d: Drive, BAS-f: Fun-seeking, BAS-r: Reward responsiveness, EFA: Exploratory factor analysis, CFA: Confirmatory factor analysis, *n*: Sample size, *SD*: Standard deviation, *α*: Cronbach’s alpha. Chi-square used to investigate sex differences and Mann-Whitney-Wilcoxon test used to investigate score differences; † Percent female.

|  | **EFA subsample** | | | | **CFA subsample** | | | |  |
| --- | --- | --- | --- | --- | --- | --- | --- | --- | --- |
|  | ***n* = 4,491** | | | | ***n* = 2,994** | | | | **Difference** |
|  | ***n*** | **Mean** | ***SD*** | ***α*** | ***n*** | **Mean** | ***SD*** | ***α*** | ***p*** |
| **Age (years)** | 4491 | 43.38 | 16.4 |  | 2994 | 43.60 | 16.14 |  | .313 |
| **Education (years)** | 4396 | 14.31 | 2.36 |  | 2943 | 14.32 | 2.33 |  | .843 |
| **Females** | 2281 | 50.8† |  |  | 1532 | 51.2 |  |  | .748 |
| **BIS** | 4454 | 20.47 | 3.36 | .76 | 2970 | 20.40 | 3.43 | .77 | .524 |
| **BAS-d** | 4458 | 10.28 | 2.52 | .80 | 2970 | 10.23 | 2.51 | .80 | .577 |
| **BAS-f** | 4457 | 11.21 | 2.30 | .72 | 2971 | 11.12 | 2.34 | .73 | .176 |
| **BAS-r** | 4457 | 16.68 | 2.07 | .70 | 2971 | 16.66 | 2.11 | .70 | .807 |

In the EFA subsample, 4,415 had complete data on BIS/BAS scales (mean age = 43.39 (SD = 16.39) years, 50.9% female). In the CFA subsample, 2,933 had complete data on BIS/BAS scales (mean age = 43.54 (SD = 16.13) years, 51.2% female).

**Supplement Table 2. Model specifications for measurement invariance.** Free: Freely estimated measure. 0: Measure set to 0. 1: Measure set to 1. 0*: Factor means were set to 0 in one group, and freely estimated in the remaining groups. For age cohort, the young group was fixed; for sex, the female group was fixed; and for longitudinal, the first wave was fixed. 1*: Residual variances were set to 1 in one group, and freely estimated in the remaining groups. For age cohort, the young group was fixed; for sex, the female group was fixed; and for longitudinal, the first wave was fixed. Equal: Measure constrained to be equal among groups. Factor variances are freely estimated since latent factor metric has been set using marker variables.

|  | **Model** | | |
| --- | --- | --- | --- |
|  | **Configural** | **Threshold** | **Loading** |
| Intercepts | 0 | 0 | 0 |
| Residual Variances | 1 | 1* | 1* |
| Factor Variances | Free | Free | Free |
| Factor Means | 0 | 0* | 0* |
| Thresholds | Free | Equal | Equal |
| Loadings | Free | Free | Equal |

**Supplement Table 3. Loading factor structure.** BIS: Behavioural inhibition system, BAS: Behavioural approach/activation system, BAS-d: drive subscale, BAS-f: fun-seeking subscale, BAS-r: reward responsiveness subscale, FFFS: Fight-flight-freeze system, WLS: Weighted least square factor, *H^2^*: Communality, *Com*: Item complexity. Bold font indicates loading ≥ .32.

|  | **BIS** | **BAS-d** | **BAS-f** | **BAS-r** | **FFFS** |  |  |
| --- | --- | --- | --- | --- | --- | --- | --- |
| **BIS** | **WLS2** | **WLS3** | **WLS1** | **WLS4** | **WLS5** | ***H^2^*** | ***Com*** |
| 1. I have very few fears compared to my friends. | .02 | -.05 | -.12 | -.03 | **.55** | .38 | 1.1 |
| 2. Criticism or scolding hurts me quite a bit. | **.70** | -.04 | -.06 | .02 | .04 | .54 | 1.0 |
| 3. I feel pretty worried or upset when I think or know somebody is angry at me. | **.75** | -.06 | .08 | -.03 | .04 | .59 | 1.0 |
| 4. If I think something unpleasant is going to happen I usually get pretty “worked-up”. | **.55** | .08 | .09 | .01 | .27 | .52 | 1.6 |
| 5. I feel worried when I think I have done poorly at something important. | **.75** | .00 | -.04 | .11 | -.10 | .57 | 1.1 |
| 6. Even if something bad is about to happen to me, I rarely experience fear or nervousness. | .01 | -.04 | .03 | .06 | **.68** | .48 | 1.0 |
| 7. I worry about making mistakes. | **.74** | .01 | -.04 | -.02 | .00 | .53 | 1.0 |
| **BAS: drive** | | | | | | | |
| 8. I go out of my way to get things I want. | -.07 | **.75** | -.01 | .03 | -.01 | .59 | 1.0 |
| 9. When I want something I usually go all-out to get it. | .01 | **.89** | -.04 | .05 | -.02 | .81 | 1.0 |
| 10. If I see a chance to get something I want I move on it right away. | -.02 | **.50** | .18 | .17 | -.06 | .53 | 1.5 |
| 11. When I go after something, I use a “no holds barred” approach. | .06 | **.49** | **.47** | -.14 | -.08 | .65 | 2.2 |
| **BAS: fun-seeking** | | | | | | | |
| 12. I’m always willing to try something new if I think it will be fun | -.15 | -.08 | **.47** | **.37** | -.14 | .50 | 2.4 |
| 13. I will often do things for no other reason than that they might be fun. | -.07 | -.01 | **.63** | .13 | -.07 | .51 | 1.1 |
| 14. I often act on the spur of the moment. | .05 | .03 | **.57** | .03 | -.04 | .38 | 1.0 |
| 15. I crave excitement and new sensations | .03 | .06 | **.75** | .00 | .03 | .60 | 1.0 |
| **BAS: reward responsiveness** | | | | | | | |
| 16. When I’m doing well at something, I love to keep at it. | .02 | .09 | -.10 | **.60** | -.05 | .38 | 1.1 |
| 17. When I get something I want, I feel excited and energised. | .02 | .14 | .05 | **.71** | .06 | .64 | 1.1 |
| 18. When I see an opportunity for something I like I get excited right away. | .14 | .21 | .28 | **.38** | .07 | .54 | 2.9 |
| 19. When good things happen to me, it affects me greatly. | .28 | -.01 | .06 | **.53** | -.05 | .47 | 1.6 |
| 20. It would excite me to win a contest. | .10 | -.01 | .19 | **.44** | .12 | .35 | 1.7 |

**Supplement Table 4. Loading structure for age, sex, and longitudinal fully invariant models.** BIS: Behavioural inhibition system, BAS: Behavioural approach/activation system, FFFS: Fight-flight-freeze system, *SE*: standard error. Please note that loadings are unstandardised.

|  | **Age cohort** | | **Sex** | | **Longitudinal** | |
| --- | --- | --- | --- | --- | --- | --- |
| **BIS** | **Estimate** | ***SE*** | **Estimate** | ***SE*** | **Estimate** | ***SE*** |
| 2. Criticism or scolding hurts me quite a bit. | 1 | 0 | 1 | 0 | 1 | 0 |
| 3. I feel pretty worried or upset when I think or know somebody is angry at me. | 1.33 | .09 | 1.16 | .06 | 1.13 | .03 |
| 4. If I think something unpleasant is going to happen I usually get pretty “worked-up”. | 1.07 | .06 | .99 | .05 | .90 | .02 |
| 5. I feel worried when I think I have done poorly at something important. | .99 | .06 | .99 | .05 | .97 | .03 |
| 7. I worry about making mistakes. | 1.01 | .06 | .92 | .05 | .92 | .03 |
| **BAS: drive** | | | | | | |
| 8. I go out of my way to get things I want. | 1 | 0 | 1 | 0 | 1 | 0 |
| 9. When I want something I usually go all-out to get it. | 1.61 | .11 | 1.66 | .10 | 1.41 | .04 |
| 10. If I see a chance to get something I want I move on it right away. | 1.13 | .07 | 1.03 | .05 | 1.11 | .03 |
| 11. When I go after something, I use a “no holds barred” approach. | 1.64 | .13 | 1.24 | .07 | 1.36 | .05 |
| **BAS: fun-seeking** | | | | | | |
| 12. I’m always willing to try something new if I think it will be fun. | 1 | 0 | 1 | 0 | 1 | 0 |
| 13. I will often do things for no other reason than that they might be fun. | 1.04 | .07 | .94 | .06 | 1.05 | .03 |
| 14. I often act on the spur of the moment. | .80 | .06 | .67 | .04 | .83 | .03 |
| 15. I crave excitement and new sensations | 1.26 | .10 | 1.04 | .07 | 1.32 | .05 |
| **BAS: reward responsiveness** | | | | | | |
| 16. When I’m doing well at something, I love to keep at it. | 1 | 0 | 1 | 0 | 1 | 0 |
| 17. When I get something I want, I feel excited and energised. | 1.82 | .16 | 1.94 | .14 | 1.99 | .08 |
| 18. When I see an opportunity for something I like I get excited right away. | 2.28 | .21 | 2.18 | .17 | 2.48 | .11 |
| 19. When good things happen to me, it affects me greatly. | 1.38 | .12 | 1.34 | .10 | 1.44 | .06 |
| 20. It would excite me to win a contest. | 1.15 | .10 | .95 | .07 | 1.24 | .05 |
| **FFFS** |  |  |  |  |  |  |
| 1. I have very few fears compared to my friends. | 1 | 0 | 1 | 0 | 1 | 0 |
| 6. Even if something bad is about to happen to me, I rarely experience fear or nervousness. | 1.34 | .11 | .93 | .07 | 1.04 | .05 |

**Supplement Table 5. Threshold structure for age, sex, and longitudinal fully invariant models.** BIS: Behavioural inhibition system, BAS: Behavioural approach/activation system, FFFS: Fight-flight-freeze system, *SE*: standard error, t1: First threshold cut-off, t2: Second threshold cut-off, t3: Third threshold cut-off. For item description, please refer to Supplement Table 4.

|  |  | **Age cohort** | | **Sex** | | **Longitudinal** | |
| --- | --- | --- | --- | --- | --- | --- | --- |
|  | **Threshold** | **Estimate** | ***SE*** | **Estimate** | ***SE*** | **Estimate** | ***SE*** |
| **BIS** | | | | | | | |
| Item 2 | t1 | -2.23 | .09 | -3.08 | .11 | -2.88 | .05 |
| Item 2 | t2 | -1.00 | .06 | -1.58 | .06 | -1.37 | .03 |
| Item 2 | t3 | .67 | .05 | .47 | .04 | .73 | .02 |
| Item 3 | t1 | -2.92 | .13 | -3.41 | .12 | -3.01 | .06 |
| Item 3 | t2 | -1.28 | .07 | -1.73 | .07 | -1.42 | .03 |
| Item 3 | t3 | 1.09 | .07 | .68 | .05 | .96 | .03 |
| Item 4 | t1 | -2.38 | .10 | -2.95 | .10 | -2.45 | .04 |
| Item 4 | t2 | -.64 | .05 | -1.07 | .05 | -.62 | .02 |
| Item 4 | t3 | 1.35 | .07 | 1.10 | .05 | 1.44 | .03 |
| Item 5 | t1 | -2.96 | .14 | -3.77 | .14 | -3.43 | .09 |
| Item 5 | t2 | -1.88 | .09 | -2.54 | .09 | -2.11 | .05 |
| Item 5 | t3 | .23 | .04 | -.10 | .04 | .26 | .02 |
| Item 7 | t1 | -2.58 | .10 | -3.03 | .10 | -2.69 | .05 |
| Item 7 | t2 | -1.17 | .06 | -1.55 | .06 | -1.24 | .03 |
| Item 7 | t3 | .88 | .05 | .57 | .04 | .82 | .02 |
| **BAS: drive** | | | | | | | |
| Item 8 | t1 | -2.30 | .10 | -1.77 | .06 | -1.94 | 0.04 |
| Item 8 | t2 | -.94 | .05 | -.36 | .04 | -.45 | .02 |
| Item 8 | t3 | 1.09 | .06 | 1.78 | .06 | 1.56 | .03 |
| Item 9 | t1 | -3.57 | .16 | -2.87 | .11 | -2.72 | .06 |
| Item 9 | t2 | -1.39 | .08 | -.49 | .06 | -.63 | .02 |
| Item 9 | t3 | 1.32 | .08 | 2.51 | .11 | 1.84 | .04 |
| Item 10 | t1 | -3.07 | .13 | -2.31 | .08 | -2.52 | .05 |
| Item 10 | t2 | -1.04 | .06 | -.35 | .04 | -.61 | .02 |
| Item 10 | t3 | 1.19 | .06 | 1.79 | .06 | 1.63 | .03 |
| Item 11 | t1 | -2.25 | .13 | -.96 | .06 | -1.27 | .04 |
| Item 11 | t2 | .30 | .07 | 1.01 | .06 | .93 | .03 |
| Item 11 | t3 | 2.71 | .15 | 2.91 | .13 | 2.89 | .08 |
| **BAS: fun-seeking** | | | | | | | |
| Item 12 | t1 | -3.77 | .19 | -3.27 | .13 | -3.19 | .07 |
| Item 12 | t2 | -2.41 | .11 | -1.78 | .07 | -1.83 | .04 |
| Item 12 | t3 | -.39 | .05 | .39 | .04 | .18 | .02 |
| Item 13 | t1 | -2.86 | .12 | -1.99 | .07 | -2.17 | .04 |
| Item 13 | t2 | -1.23 | .06 | -.45 | .04 | -.54 | .02 |
| Item 13 | t3 | .85 | .06 | 1.53 | .06 | 1.43 | .03 |
| Item 14 | t1 | -2.38 | .10 | -1.61 | .06 | -1.66 | .03 |
| Item 14 | t2 | -.77 | .05 | -.19 | .03 | -.22 | .02 |
| Item 14 | t3 | 1.10 | .06 | 1.53 | .05 | 1.33 | .03 |
| Item 15 | t1 | -2.87 | .18 | -1.67 | .06 | -1.83 | .04 |
| Item 15 | t2 | -.90 | .08 | .03 | .04 | .09 | .02 |
| Item 15 | t3 | 1.25 | .09 | 1.91 | .08 | 1.96 | .05 |
| **BAS: reward responsiveness** | | | | | | | |
| Item 16 | t1 | -3.13 | .20 | -3.28 | .16 | -3.22 | .07 |
| Item 16 | t2 | -2.35 | .13 | -2.39 | .10 | -2.39 | .05 |
| Item 16 | t3 | -.51 | .04 | -.37 | .03 | -.31 | .01 |
| Item 17 | t1 | -3.94 | .24 | -4.07 | .17 | -4.02 | .10 |
| Item 17 | t2 | -2.83 | .16 | -2.87 | .11 | -2.61 | .06 |
| Item 17 | t3 | -.43 | .05 | -.15 | .05 | .00 | .02 |
| Item 18 | t1 | -4.05 | .25 | -3.69 | .17 | -3.74 | .10 |
| Item 18 | t2 | -2.08 | .13 | -1.71 | .09 | -1.62 | .05 |
| Item 18 | t3 | .67 | .07 | 1.04 | .06 | 1.30 | .04 |
| Item 19 | t1 | -3.36 | .19 | -3.55 | .17 | -3.62 | .09 |
| Item 19 | t2 | -2.11 | .10 | -2.10 | .08 | -2.01 | .04 |
| Item 19 | t3 | .03 | .04 | .31 | .04 | .38 | .02 |
| Item 20 | t1 | -2.93 | .13 | -2.35 | .08 | -2.45 | .04 |
| Item 20 | t2 | -1.90 | .08 | -1.47 | .05 | -1.48 | .03 |
| Item 20 | t3 | -.04 | .04 | .12 | .03 | .30 | .02 |
| **FFFS** | | | | | | | |
| Item 1 | t1 | -1.80 | .08 | -1.96 | .07 | -1.69 | .04 |
| Item 1 | t2 | .14 | .05 | -.05 | .04 | .17 | .02 |
| Item 1 | t3 | 1.89 | .09 | 1.71 | .07 | 1.92 | .04 |
| Item 6 | t1 | -2.32 | .13 | -1.90 | .07 | -1.77 | .03 |
| Item 6 | t2 | -.46 | .06 | -.52 | .04 | -.22 | .02 |
| Item 6 | t3 | 1.39 | .09 | .89 | .05 | 1.21 | .03 |

**Supplement Table 6. Age and sex inter-factor correlations.** Correlations taken from the fully invariant models. BIS: Behavioural inhibition, BAS: Behavioural approach/activation, BAS-d: Drive, BAS-f: Fun-seeking, BAS-r: Reward responsiveness, FFFS: Fight-flight-freeze. Bold font indicates *r* ≥ .32.

|  |  | **BIS** | **BAS-d** | **BAS-f** | **BAS-r** | **FFFS** |
| --- | --- | --- | --- | --- | --- | --- |
| **Age** | **Young** | | | | | |
|  | **BIS** | 1 |  |  |  |  |
|  | **BAS-d** | -.13 | 1 |  |  |  |
|  | **BAS-f** | -.12 | **.57** | 1 |  |  |
|  | **BAS-r** | .16 | .31 | **.33** | 1 |  |
|  | **FFFS** | **.70** | **-.44** | **-.41** | -.05 | 1 |
|  | **Midlife** | | | | | |
|  | **BIS** | 1 |  |  |  |  |
|  | **BAS-d** | -.05 | 1 |  |  |  |
|  | **BAS-f** | .04 | **.52** | 1 |  |  |
|  | **BAS-r** | .25 | .27 | .28 | 1 |  |
|  | **FFFS** | **.40** | **-.32** | -.29 | -.03 | 1 |
|  | **Old age** | | | | | |
|  | **BIS** | 1 |  |  |  |  |
|  | **BAS-d** | .10 | 1 |  |  |  |
|  | **BAS-f** | .17 | **.68** | 1 |  |  |
|  | **BAS-r** | .31 | **.32** | **.37** | 1 |  |
|  | **FFFS** | .24 | **-.32** | **-.37** | -.09 | 1 |
| **Sex** | **Females** | | | | | |
|  | **BIS** | 1 |  |  |  |  |
|  | **BAS-d** | .04 | 1 |  |  |  |
|  | **BAS-f** | .08 | **.74** | 1 |  |  |
|  | **BAS-r** | .31 | **.38** | **.41** | 1 |  |
|  | **FFFS** | **.51** | -.27 | -.30 | -.03 | 1 |
|  | **Males** | | | | | |
|  | **BIS** | 1 |  |  |  |  |
|  | **BAS-d** | -.02 | 1 |  |  |  |
|  | **BAS-f** | .03 | **.87** | 1 |  |  |
|  | **BAS-r** | .24 | **.43** | **.51** | 1 |  |
|  | **FFFS** | **.38** | **-.38** | **-.35** | -.11 | 1 |

**Supplement Table 7. Longitudinal inter-factor correlations.** Correlations taken from the fully invariant models for time. BIS: Behavioural inhibition, BAS: Behavioural approach/activation, BAS-d: drive, BAS-f: fun-seeking, BAS-r: reward responsiveness, FFFS: Fight-flight-freeze. Bold font indicates *r* ≥ .32.

|  | **BIS** | **BAS-d** | **BAS-f** | **BAS-r** | **FFFS** |
| --- | --- | --- | --- | --- | --- |
| **Wave 1** | | | | | |
| **BIS** | 1 |  |  |  |  |
| **BAS-d** | -.02 | 1 |  |  |  |
| **BAS-f** | .04 | **.70** | 1 |  |  |
| **BAS-r** | .28 | **.36** | **.36** | 1 |  |
| **FFFS** | **.49** | **-.34** | -.28 | -.03 | 1 |
| **Wave 2** | | | | | |
| **BIS** | 1 |  |  |  |  |
| **BAS-d** | .01 | 1 |  |  |  |
| **BAS-f** | .04 | **.72** | 1 |  |  |
| **BAS-r** | .28 | **.37** | **.36** | 1 |  |
| **FFFS** | **.50** | **-.35** | -.30 | -.04 | 1 |
| **Wave 3** | | | | | |
| **BIS** | 1 |  |  |  |  |
| **BAS-d** | .08 | 1 |  |  |  |
| **BAS-f** | .16 | **.64** | 1 |  |  |
| **BAS-r** | **.35** | **.41** | **.39** | 1 |  |
| **FFFS** | **.51** | **-.31** | -.27 | -.03 | 1 |
| **Wave 4** | | | | | |
| **BIS** | 1 |  |  |  |  |
| **BAS-d** | .05 | 1 |  |  |  |
| **BAS-f** | .12 | **.65** | 1 |  |  |
| **BAS-r** | **.32** | **.35** | **.35** | 1 |  |
| **FFFS** | **.51** | **-.32** | -.30 | -.03 | 1 |

# Supplementary Figures

**Supplement Figure 1. BIS/BAS 4-factor model**

Loadings < .32 have been omitted. Dashed lines represent lower double loadings. BIS: Behavioural inhibition system, BAS: Behavioural approach/activation system, BAS-d: Drive, BAS-f: Fun-seeking, BAS-r: Reward responsiveness, χ^2^: Chi-square, TLI: Tucker Lewis index, RMSEA: Root mean square error of approximation, CI: Confidence interval, BIC: Bayesian information criterion.


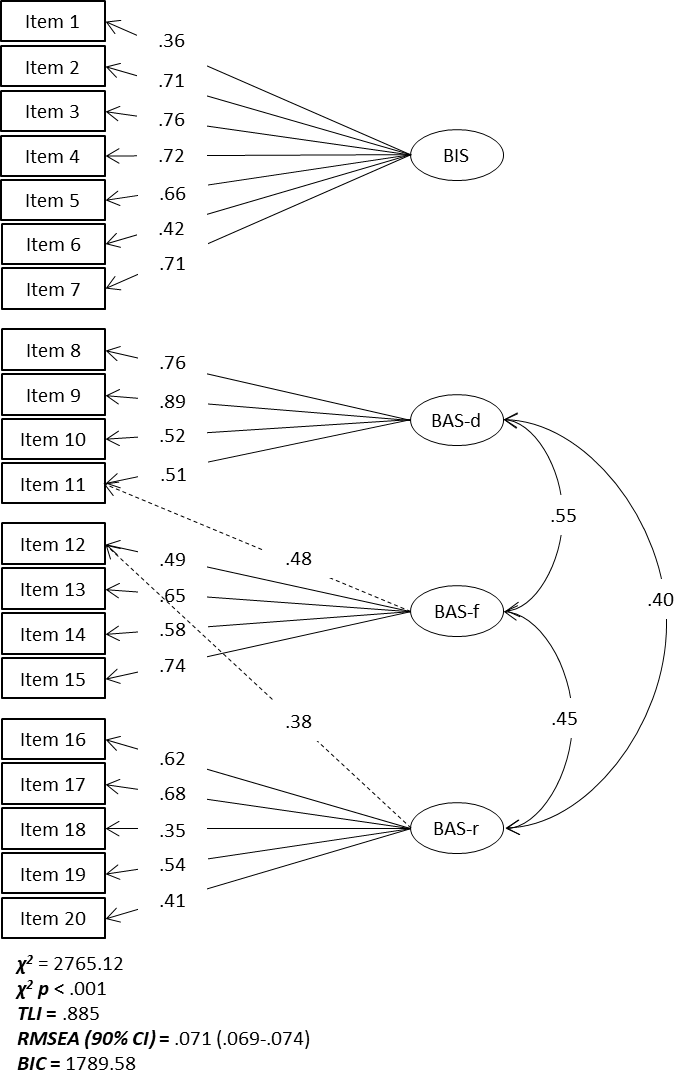


**Supplement Figure 2. BIS/BAS/FFFS 6-factor model**

Loadings < .32 have been omitted. Dashed lines represent lower double loadings. BIS: Behavioural inhibition system, BAS: Behavioural approach/activation system, BAS-d: Drive, BAS-f: Fun-seeking, BAS-r: Reward responsiveness, FFFS: Fight-flight-freezing χ^2^: Chi-square, TLI: Tucker Lewis index, RMSEA: Root mean square error of approximation, CI: Confidence interval, BIC: Bayesian information criterion.


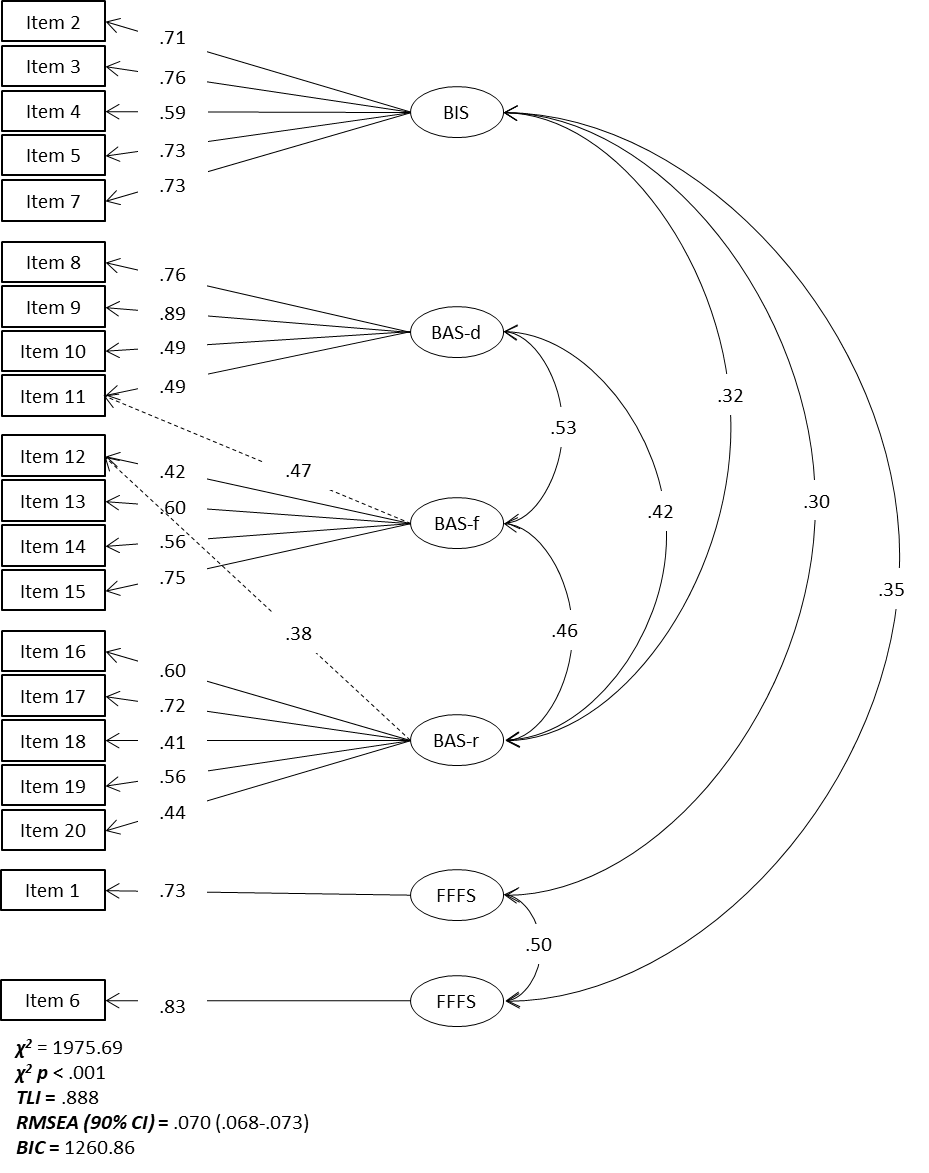


**Supplement Figure 3. Associations between BIS/BAS, EPQ-r, PANAS, and GADS in females**

**
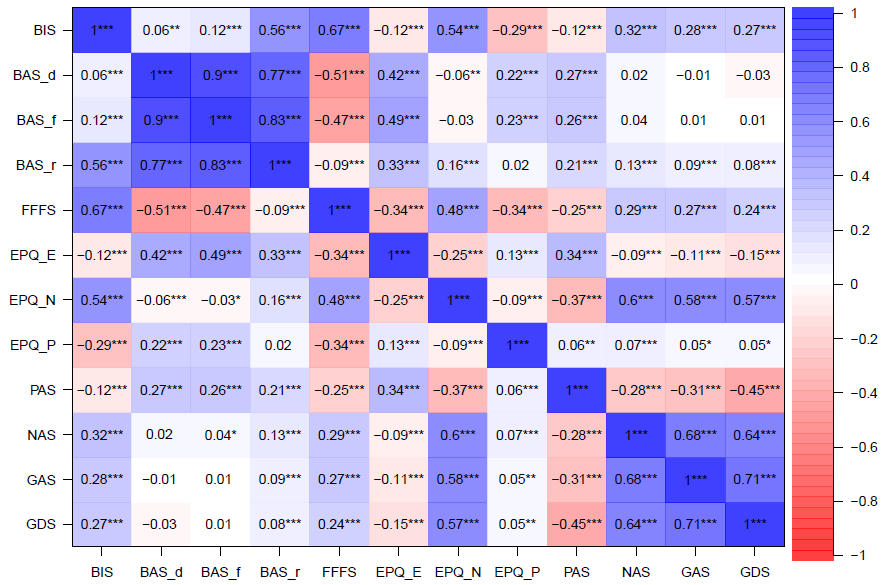
**BIS: Behavioural inhibition system, BAS: Behavioural activation system, BAS_d: Drive subscale, BAS_f: Fun-seeking subscale, BAS_r: Reward responsiveness subscale, FFFS: Fight-flight-freeze system, EPQ: Eysenck personality questionnaire, EPQ_E: Extraversion scale, EPQ_N: Neuroticism scale, EPQ_P: Psychoticism scale, PAS: Positive affect scale, NAS: Negative affect scale, GAS: Goldberg anxiety scale, GDS: Goldberg depression scale. Far right bar shows value of correlation. *: *p* ≤ .05, **: *p* ≤ .01, ***: *p* ≤ .001

**Supplement Figure 4. Associations between BIS/BAS, EPQ-r, PANAS, and GADS in males**

**
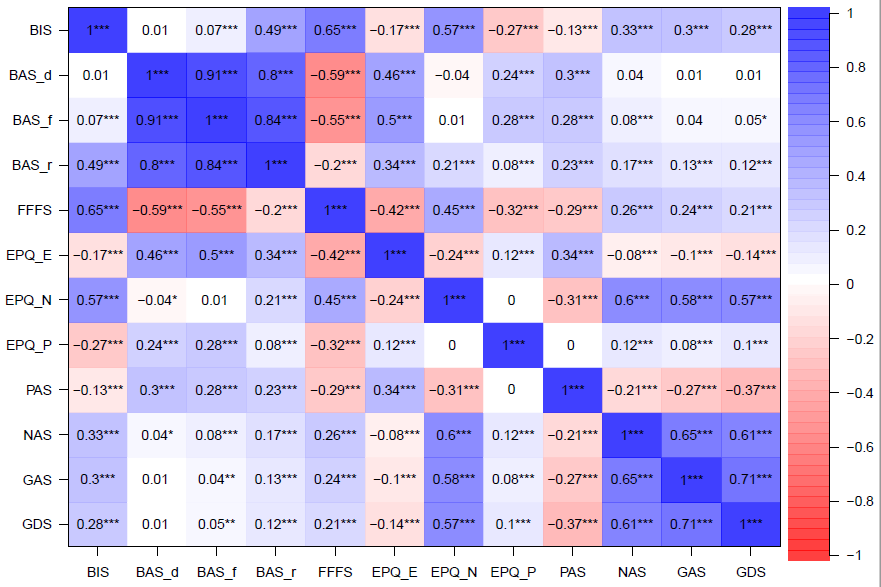
**BIS: Behavioural inhibition system, BAS: Behavioural activation system, BAS_d: Drive subscale, BAS_f: Fun-seeking subscale, BAS_r: Reward responsiveness subscale, FFFS: Fight-flight-freeze system, EPQ: Eysenck personality questionnaire, EPQ_E: Extraversion scale, EPQ_N: Neuroticism scale, EPQ_P: Psychoticism scale, PAS: Positive affect scale, NAS: Negative affect scale, GAS: Goldberg anxiety scale, GDS: Goldberg depression scale. Far right bar shows value of correlation. *: *p* ≤ .05, **: *p* ≤ .01, ***: *p* ≤ .001

**References**

Muthén, L.K., and Muthén, B.O. (1998-2011). *Mplus user's guide.* Los Angeles, CA.

Revelle, W. (2018). "Procedures for personality and psychological research ". 1.8.12 ed. (Evanston, Illinois, USA: Northwestern University).

Rosseel, Y. (2012). lavaan: An R package for structural equation modeling. *Journal of Statistical Software* 48**,** 1-36.
